# Flavonoids Derived from the Roots of *Lespedeza bicolor* Inhibit the Activity of SARS-CoV Papain-like Protease

**DOI:** 10.3390/plants13233319

**Published:** 2024-11-26

**Authors:** Hyun Sim Woo, Kon Ho Lee, Ki Hun Park, Dae Wook Kim

**Affiliations:** 1 Forest Bioresources Department, Baekdudaegan National Arboretum, Bonghwa-gun 36209, Republic of Korea; whs0428@koagi.or.kr; 2 Department of Microbiology, School of Medicine, Gyeongsang National University, Jinju 52727, Republic of Korea; lkh@ac.kr; 3 Division of Applied Life Science (BK21 Four), IALS, Gyeongsang National University, Jinju 52828, Republic of Korea; khpark@gnu.ac.kr

**Keywords:** SARS-CoV PLpro, *Lespedeza bicolor*, pterocarpan, PLpro inhibitor

## Abstract

Despite the now infamous coronavirus disease outbreaks caused by severe acute respiratory syndrome coronavirus (SARS-CoV), this virus continues to be a threat to the global population. Although a huge research effort has targeted SARS-CoV, no report exists regarding natural small molecules targeting one of its key enzymes, papain-like protease (PLpro). In this study, nine flavonoids displaying SARS-CoV PLpro inhibitory activity were isolated from the root bark of *Lespedeza bicolor*. The compounds were identified as erythrabyssin II (**1**), lespebuergine G4 (**2**), 1-methoxyerythrabyssin II (**3**), bicolosin A (**4**), bicolosin B (**5**), bicolosin (**6**), xanthoangelol (**7**), (±)-lespeol (**8**), and parvisoflavanone (**9**). Most compounds (**1**–**4** and **6**–**8**) inhibited SARS-CoV PLpro activity in a dose-dependent manner, with their *K*_i_s ranging from 5.56 to 75.37 μM. The structure–activity analysis of pterocarpans (**1**–**6**) showed that activity was enhanced by C1-OCH_3_, but it was reduced by C8-CH_3_. A mechanistic analysis revealed that all inhibitors were noncompetitive. Some of the key compounds isolated in this study are pterocarpans, which are abundantly present in the Leguminosae family. Overall, a rich source of SARS-CoV papain-like protease inhibitors was identified in this study.

## 1. Introduction

The SARS-CoV-2 coronavirus, along with the SARS-CoV and MERS-CoV coronaviruses, is a zoonotic coronavirus that belongs to the Coronaviridae family and class B of the Betacoronavirus genus [1]. These coronaviruses are a highly contagious zoonotic RNA virus that causes fatal respiratory illness. Coronavirus disease (COVID-19), a respiratory illness, is caused by SARS-CoV-2 and was first reported in December 2019 [2]. The World Health Organization reported 9,129,146 confirmed cases, which resulted in 473,797 deaths with a fatality rate of 5.18% worldwide (as of 25 June 2023). The fatality rate for SARS-CoV infection is 5.18%. Although the global scientific community successfully created several authorized and efficacious COVID-19 vaccines, effective treatment for COVID-19 is still lacking due to heterogenous symptoms and newly emerging strains; therefore, there is an urgent need for effective therapeutic drugs.

SARS-CoV can be controlled by targeting numerous critical steps involved in its replication. The cleavage of a multidomain viral polyprotein into 16 mature components, which are necessary for the synthesis of viral RNA 41, is an early and essential process that occurs during the replication of SARS-CoV [3]. The viral envelope consists of four structural proteins: a membrane protein, an envelope protein, a nucleoprotein, and a spike protein. Two big polyproteins, pp1a (4405 amino acids) and Pp1ab (7096 amino acids), are used by the CoV-2 genomic (+) sense ssRNA to generate 15 nonstructural proteins (Nsps) in order to accomplish replication [4]. Two cysteine proteases, a papain-like protease (PLpro) and a 3C-like protease (3CLpro), [5] catalyze their own release and free other nonstructural proteins (NSPs) from the polyprotein in this key maturation process. Potential functions of PLpro beyond viral peptide cleavage have been proposed by recent structural and functional studies, including deubiquitination, deISGylation, and immune response evasion [6,7,8]. The multiple roles of PLpro in viral replication and pathogenesis indicate that it could serve as a promising target for antiviral therapeutics. As potential inhibitors of SARS-CoV PLpro, a number of different compounds have been developed. However, there are currently no known natural compounds that have inhibitory activity against SARS-CoV PLpro.

*Lespedeza bicolor Turcz*(Fabaceae) is distributed across East Asia and North America and has been used as a traditional medicine to treat athlete’s foot, cough, skin ailments, and vascular disease. The vermicide and dermatosis treatment properties of *L. bicolor* are particularly well known. Its primary bioactive components include organic acids, stigmasterols, terpenes, alkaloids, isoflavanones, pterocarpans, and flavonoids [9,10,11,12]. Among these, several compounds exhibit antibacterial, antifungal [13], and antioxidative [14] activities. By using quantitative structure–activity relationship (QSAR) assays, a recent study showed that prenylated pterocarpans derived from *L. bicolour* exhibited significant virucidal activity [15]. Antidiabetic effects have been reported by reducing blood glucose levels and improving insulin sensitivity through the use of *L. bicolour* extracts, which contain a variety of compounds including genistein, quercetin, catechin, and naringin [9]. During an intensive study on biologically active metabolites from *L. bicolor*, we found that its methanol extract significantly inhibited SARS-CoV PLpro activity.

In this investigation, we extracted nine phenolic compounds from *L. bicolour* roots that target SARS-CoV PLpro. The inhibitory properties of each isolated compound against PLpro were assessed separately. Their inhibition mechanisms were elucidated using the Lineweaver–Burk, Dixon, Hanse–Woolf, and Woolf–Hofstee plots. To the best of our knowledge, this is the first study to investigate the inhibitory activity of compounds derived from the roots of *L. bicolor* against proteolytic enzymes.

## 2. Results

### 2.1. Isolation of Flavonoids and Their Inhibitory Effect on SARS-CoV PLpro Activity

**Figure 1:**
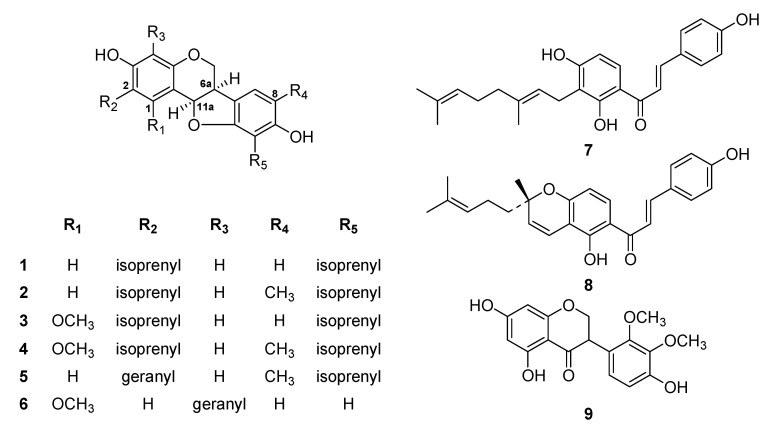
Chemical structures of compounds **1**–**9** isolated from *L. bicolor*.

During preliminary screening, the methanol extract of *L. bicolor* roots significantly inhibited SARS-CoV PLpro activity. The methanol extract was fractionated using successive silica gel and reverse phase column chromatography. Totally, nine flavonoids (**1**–**9**) were isolated and identified as the known species erythrabyssin II (**1**) [16], lespebuergine G4 (**2**) [17], 1-methoxyerhtrabyssin II (**3**) [18], bicolosin A (**4**), bicolosin B (**5**), bicolosin C (**6**) [12], xanthoangelol (**7**), (±)-lsepeol (**8**) [19], and parvisoflavanone (**9**) based on spectroscopic data and by comparing with the findings of previous studies (Figure 1) [20].

### 2.2. Expression of SARS-CoV PLpro in E. coli

The SARS-CoV PLpro (residues 154-1855) was successfully expressed in a soluble form in *E. coli* and subsequently purified using gel filtration, ion-exchange, and nickel affinity chromatography techniques. The expression of PLpro was confirmed using MALDI-TOF mass spectrometry. The PLpro protein was separated as a monomer through gel filtration. The histidine tag used for purification was removed via TEV cleavage to avoid any interference in the enzyme activity. The purified PLpro effectively cleaved the fluorogenic peptide substrate, as described in the inhibition assay (Supplementary Materials).

### 2.3. SARS-CoV PLpro Activity Assay

**Figure 2:**
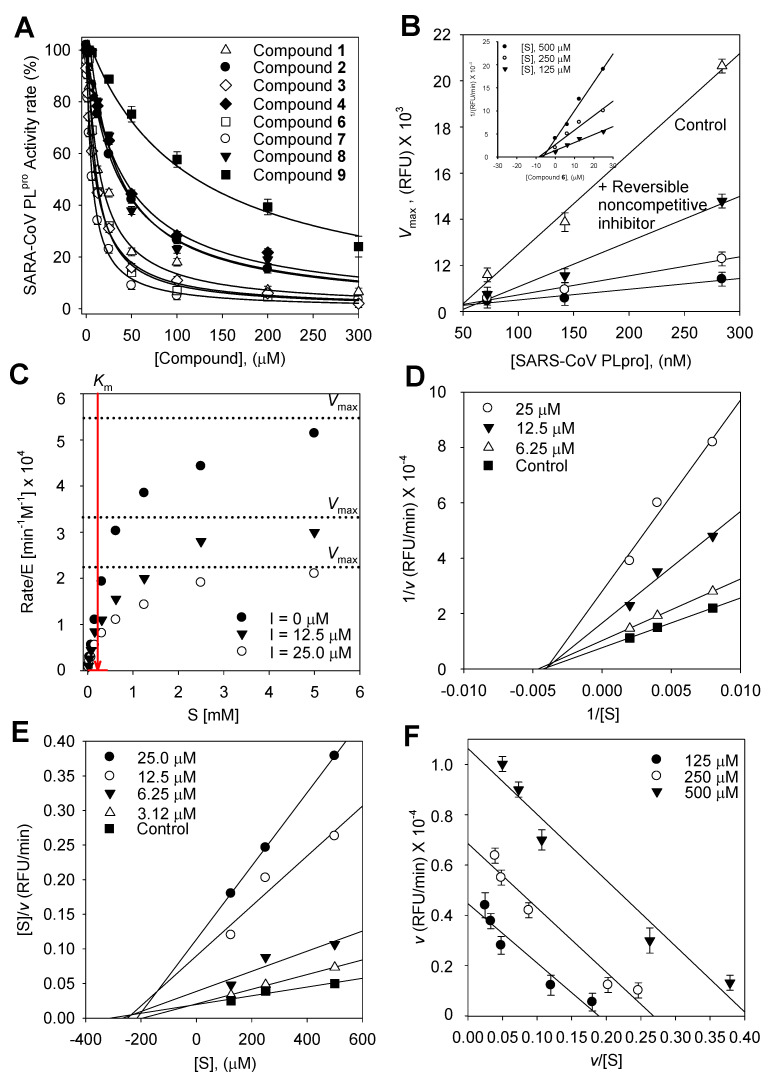
(**A**) Effects of isolated compounds **1**–**9** on SARS-CoV PLpro used for the hydrolysis of Z-RLRGG-AMC. (**B**) Catalytic activity of SARS-CoV PLpro as a function of enzyme concentration at different concentrations of compound **6** (▽, 0 μM; ▼, 6.25 μM; ○, 12.5 μM; ●, 25.0 μM). (Insert) The Dixon plot for compound **6** to determine its inhibition constant *K_i_*, and the inhibitory effect of **6** on the enzymatic hydrolysis of Z-RLRGG-AMC displayed as the (**C**) hyperbolic data, (**D**) Lineweaver–Burk plot, (**E**) Hanse–Woolf plot, and (**F**) Woolf–Hofstee plot.

We used an in vitro fluorescence assay to measure SARS-CoV PLpro activity both with and without the test compounds in order to examine the relative inhibitory effects of the nine isolated compounds (**1**–**9**) against SARS-CoV PLpro. The hydrolytic activity of SARS-CoV PLpro was determined using a substrate in 20 mM Tris buffer at pH 8 and 37 °C under steady state conditions. Plotting the initial rates adjusted for the enzyme concentration (1.4 × 10^−7^ M) against the substrate concentration (0–5 mM) and fitting the hyperbolic data using non-linear regression with the Michaelis–Menten model (Figure 2C) allowed for the determination of the apparent Michaelis–Menten constant (*K*_m_ = 250 ± 0.9 μM).

### 2.4. Enzyme Kinetic Analysis

**Figure 3:**
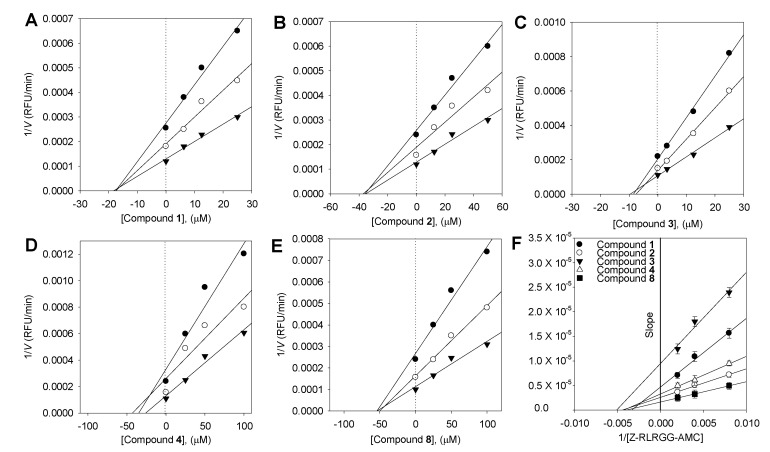
Dixon plots for the inhibition of compounds **1**–**4** and **8** on SARS-CoV PLpro for the hydrolysis of Z-RLRGG-AMC: compounds **1**–**4** (**A**–**D**), and compound **8** (**E**). The following substrate concentrations were used: 500 μM (▼), 250 μM (○), and 125 μM (●). (**F**) Slope replots: compound **1** (●), compound **2** (○), compound **3** (▼), compound **4** (△), and compound **8** (■).

To describe the suppression of Z-RLRGG-AMC hydrolysis, the kinetic tests were conducted again with varying concentrations of compounds (**1**–**4** and **6**–**9**). The addition of **1**–**4** and **6**–**9** decreased the *V*_max_, but the apparent Michaelis–Menten constant (*K*_m_) remained unaffected. The hyperbolic data and Lineweaver–Burk, Dixon, Hanes–Woolf, and Woolf–Hofstee plots [18] following inhibition by **6** are depicted as representative examples (Figure 2). The *K*_i_ values of all flavonoids were measured using Dixon plots (Figure 3).

## 3. Discussion

Although various studies have explored the biochemical and structural aspects of PLpro and inhibitors targeting PLpro, natural inhibitors of this enzyme have not been reported. In the present study, we isolated compounds from *L. bicolor* and their structures were identified using NMR methods (^1^H-NMR, ^13^C-NMR, 1H-1H COSY, HSQC, and HMBC) and HRMS. Using the methanol extract of *L. bicolor* roots, we purified compounds displaying inhibitory effects against PLpro using activity-guided fractionation. Repeated chromatography of this extract yielded six pterocarpans (**1**–**6**), two chalcones (**7**–**8**), and one isoflavone (**9**). Pterocarpans were formed by the oxidative ring cyclization of isoflavones catalyzed by a cytochrome P-450-dependent enzyme. This class of natural products is almost exclusively restricted to the Leguminosae family, implying that Leguminosae may constitute a rich source of compounds with activity against SARS-CoV PLpro. Importantly, we found that the most prevalent secondary metabolites of *L*. *bicolor* were pterocarpans.

^1^H-^1^H COSY of the isolated compounds **1**–**8** indicated the presence of an isoprenyl group and a main skeleton in each compound. The positions of the isoprenyl groups and quaternary carbons in each compound were identified by performing a ^1^H-^13^C HMBC experiment, which shows long-range connectivity between protons and carbons. The coupling constant between H-6a and H-11a [J = 6.4 Hz, indicating an axial–equatorial arrangement] agreed with the finding that pterocarpans are found in nature only in the 6a, 11a-*cis* configuration [21]. The absolute configuration of the pterocarpan at C-6a was assigned as *R* based on its negative optical rotation and positive Cotton effect [22,23] at 285 nm (additional information). For example, the most potent pterocarpan, pterocarpan **6**, showed an [α]$\begin{array}{c} 20\\ D \end{array}$ = −122 (c 0.21, MeOH) + 20.8 (at 286 nm) Cotton effect. By comparing the physical and spectral data of these isolated polyphenols with those of known compounds, we identified the nine isolated species as erythrabyssin II (**1**), lespebuergine G4 (**2**), 1-methoxyerhtrabyssin II (**3**), bicolosin A (**4**), bicolosin B (**5**), bicolosin C (**6**), xanthoangelol (**7**), (±)-lsepeol (**8**), and parvisoflavanone (**9**).

All the tested compounds displayed a dose-dependent inhibitory effect on SARS-CoV PLpro activity (IC_50_ = 9.3–217.4 μM, Table 1). In the pterocarpan system, geranylation (**5**, IC_50_ = 217.4 μM) clearly led to a significant decrease in inhibition potency when compared with isoprenylation (**2**, IC_50_ = 32.9 μM). Thus, steric constraints may play a role in binding. Furthermore, pterocarpans (**1**–**6**) demonstrate less significant positional preferences against C8-CH_3_ for C1-OCH_3_ functionalities. Methylation at C8 resulted in reduced activity, as observed through comparisons of **1** (IC_50_ = 18.22 μM) vs. **2** (IC_50_ = 32.97 μM) and **3** (IC_50_ = 11.21 μM) vs. **4** (IC_50_ = 43.26 μM). In contrast, the presence of a methoxy group on C1 (**3**, IC_50_ = 11.2 μM) produced a 2-fold higher inhibition than its demethoxylated analog, **1** (IC_50_ = 18.2 μM). In the case of chalcones, ring closure of the geranyl group onto C4-OH to form hexacyclic structures resulted in 8-fold lower potency: **7** (IC_50_ = 6.3 μM) vs. **8** (IC_50_ = 50.1 μM).

All the inhibitors exhibited a similar relationship with enzyme activity and concentration. The inhibition of SARS-CoV PLpro activity by **6**, the most potent pterocarpan (*K*_i_ = 9.06 μM), is shown in Figure 2. An increase in the concentration of inhibitors resulted in a reduction in the residual enzyme activity. Plots of residual enzyme activity versus enzyme concentration at different concentrations of compound **6** showed several straight lines with a *y*-intercept of 0, indicating that **6** is a reversible inhibitor of SARS-CoV PLpro. The Lineweaver–Burk, Dixon, Hanse–Woolf, and Woolf–Hofstee plots obtained from the kinetic data in the presence of **6** indicated the noncompetitive inhibition of SARS-CoV PLpro. For instance, compound **6** reduced the *V*_max_, but it exhibited no effect on the Michaelis–Menten constant (*K*_m_). The *K*_i_ values of all compounds were measured using a Dixon plot (Figure 2B insert). In a plot of [S]/*v* versus [S] using Equation (2), increasing the concentration of **6** resulted in several lines with a common intercept on the [S] axis but with different gradients (Figure 2E), reaffirming the noncompetitive mode of enzyme inhibition [24].

[S]/*v* = 1/*V*_max_[S] + *Ks*/*V*_max_ (1)

A plot of *v* versus *v*/[S] at differing concentrations of **6** was fitted to Equation (3) to determine the variation in *V*_max_ as a function of the substrate.

(2) $$V=-K_{\mathrm{Sapp}}\;\frac{v}{\left[S\right]}+V_{\max}$$

The *V*_max_ decreased as the concentration of **6** increased. Consistent with noncompetitive inhibition, each line (corresponding to a particular inhibitor concentration) was parallel.

Until the present date, none of the natural products have been reported to demonstrate inhibitory effects against the SARS-CoV proteolytic enzymes PLpro and 3CLpro, which are essential for viral replication. Herein, we present the first examples of natural SARS-CoV PLpro inhibitors derived from *L. bicolor*, a plant of the Leguminosae family widely used in traditional medicine. Since the Leguminosae family is known to be rich in pterocarpans (a key structural component of some of our most potent inhibitors), our data indicate that other Leguminosae members may also contain active species that could target PLpro. The inhibitors identified in this study exhibited non-competitive enzyme inhibition, and their IC_50_ values were in the low micromolar range. Thus, they are candidate lead compounds for the discovery and development of drugs targeting SARS-CoV. Our study also elucidated some aspects of the structure–activity relationship (SAR) of these species, which would provide direction for further development.

## 4. Materials and Methods

### 4.1. General Apparatus and Chemicals

All reagent-grade chemicals were purchased from Sigma Chemical Co. (St. Louis, MO, USA). Chromatographic separation was performed by thin-layer chromatography, using commercially available glass plates pre-coated with silica gel (E. Merck Co., Darmstadt, Germany), and the products were visualized under UV at 254 and 366 nm or sprayed with a 10% H_2_SO_4_ staining reagent. Silica gel (230–400 mesh, Merck), RP-18 (ODS-A, 12 nm, S-150 μM, YMC), and Sephadex LH-20 (Cytiva) were used for column chromatography. ^1^H and ^13^C NMR along with 2D NMR data were obtained on a Bruker AM 300 or 500 MHz spectrometer using CDCl_3_ with tetramethylsilane (TMS) as an internal standard. EIMS and HREIMS data were collected on a Jeol JMS-700 spectrometer. Melting points were measured on a Thomas Scientific Capillary Melting Point Apparatus and were uncorrected. UV spectra were measured on a Beckman DU650 spectrophotometer. Infrared (IR) spectra were recorded on a Bruker IFS66 infrared Fourier transform spectrophotometer (on KBr disks). CD spectra were recorded on a JASCO J-715 spectropolarimeter, and optical rotations were measured on a Perkin-Elmer 343 polarimeter.

### 4.2. Plant Material

The root bark of *L. bicolor* was collected at Geumsan in Daejeon, South Korea, in January 2010 and identified by Prof. Jae-Hong Pak. A voucher specimen (KHPark 010711) of this raw material was deposited at the herbarium of Kyungpook National University (KNU).

### 4.3. Extraction and Isolation of Bioactive Compounds

The air-dried root bark (1.5 Kg) of *L. bicolor* was chopped and extracted with MeOH (20 L) at room temperature for a week. The combined filtrate was concentrated in vacuo to yield a dark brown gum (65 g). This extract was fractionated by column chromatography on silica gel (10 × 75 cm, 230–400 mesh, 760 g) and eluted using *n*-hexane/EtOAc (90:10 → 80:20 → 70:30 → 60:40 → 50:50 → 40:60 → 20:80 →0:100, 1500 mL each) to yield six fractions (A–F). Subfractions C and D (12 g), exhibiting potent SARS-CoV PLpro inhibition, were rechromatographed over a silica gel column (4 × 50 cm, 230–400 mesh, 270 g) using a hexane/acetone gradient [60:1 (500 mL), 40:1 (500 mL), 20:1 (450 mL), 10:1 (900 mL), 5:1 (900 mL), 3:1 (350 mL), 1:1 (500 mL)] to afford nine subfractions (F1-9). Subfraction F4 (720 mg), enriched with **3**, **4**, and **9**, was chromatographed on silica gel (2.5 × 40 cm, 150 g) using a hexane/acetone gradient to yield **3** (13 mg), **4** (33 mg), and **9** (10 mg). Subfraction F5 (1.2 g) was separated by column chromatography (2.0 × 50 cm) on silica gel (230–400 mesh, 130 g) using *n*-hexane/EtOAc (20:1 → 1:1) to yield 39 subfractions. Subfractions F4.12-17, enriched with **1** and **2**, were combined (240 mg) and further purified by silica gel flash CC to yield **1** (30 mg) and **2** (22 mg). F6 (860 mg) was applied to a silica gel column (2.5 × 60 cm, 230–400 mesh, 170 g) and chromatographed using a chloroform/EtOAc gradient (20:1 → 1:1) to yield 13 subfractions (F6.1-13). Subfractions F6.10-12, enriched with **5** and **8**, were combined (210 mg), purified by reversed-phase CC (ODS-A, 12 nm, S-150 μM), and eluted with CH_3_OH:H_2_O (4:1) to yield **5** (14 mg) and **8** (8 mg). Subfraction F7 (920 mg) was rechromatographed on a silica gel column (2.0 × 15 cm, 230–400 mesh, 200 g), using chloroform/acetone (10:1 → 1:1), to yield 20 fractions. Fractions 13–16, enriched with **6** and **7**, were combined (170 mg) and chromatographed on a Sephadex LH-20 column (1.5 × 50 cm, 50 g) to afford **6** (11 mg) and **7** (23 mg).

### 4.4. Expression and Purification of SARS-CoV PLpro in E. coli

The gene encoding PLpro (945 bp) was amplified using the plasmid pSARS-REP (SARS-CoV ulbani strain, Genbank AY278741), forward primer 5′-GCGGGATCCGAGGTTAAGACTATAAAAGTGTTC-3′, and reverse primer 5′-GCGCTCGAGTTACTTGATGGTTGTAGTGTAAGA′ (BamHI and XhoI sites are underlined). The gene was ligated into a PCR-TOPO (Invitrogen, Carlsbad, CA, USA) and insertion was confirmed by DNA sequencing with T7 forward and T7 terminal reverse primers. Subsequently, the correct gene fragment was transferred into a pPRoEx HT expression vector (Invitrogen), which was then used to transform DH5α competent cells. The expression plasmid was constructed for the recombinant PLpro to carry a His tag and a TEV site at the N-terminus. Correct clones of PLpro in the pPRoEX HT vector were identified and verified by PCR and restriction digestion with BamHI and XhoI. The pPRoEx HT plasmid harboring the PLpro gene was transformed into an *E. coli* strain, BL21(DE3) (Novagen, Madison, WI, USA), for protein expression. A 10 mL aliquot of an overnight culture was seeded into 1000 mL of a fresh Luria–Bertani (LB) medium containing 50 mg/mL ampicillin, and the cells were grown to an OD_600 nm_ of 0.6 at 37 °C. The cells were cooled down in ice for 30 min, and protein expression was induced for 5 h using 0.4 mM isopropyl β-D-1-thiogalactopyranoside (IPTG) at 30 °C. The cells were harvested by centrifugation at 6000 rpm for 6 min at 4 °C. The harvested cells were washed twice in phosphate-buffered saline. The cell pellet was directly used for protein isolation or stored at −80 °C until use.

The cell pellet was suspended in a binding buffer (50 mM NaH_2_PO_4_, pH 8.0; 500 mM NaCl; 5 mM imidazole; and 5 mM β-mercaptoethanol), and the cells were disrupted by sonication. After centrifugation at 15,000 rpm for 1 h, the clear supernatant was collected, filtered (Qualitative filter paper, Advantec, Japan), and applied onto a column of Nickel Sepharose 6 Fast Flow (GE Healthcare, Uppsala, Sweden) beads pre-equilibrated with the binding buffer. First, the column was washed with 20 column volumes of the binding buffer and then with 2 column volumes of a washing buffer (50 mM Tris-HCl, pH 8.0; 500 mM NaCl; 30 mM imidazole). The recombinant PLpro proteins were eluted using the elution buffer (50 mM Tris-HCl, pH 8.0; 100 mM NaCl; and 300 mM imidazole). The PLpro eluted from the nickel column was further purified by ion-exchange chromatography using a salt gradient with a SOURCE 15Q column (GE Healthcare, Piscataway, NJ, USA) in 50 mM Tris-HCl, pH 8.5, and 2 mM DTT. The PLpro was finally purified using size exclusion chromatography with a Superdex 200 column (GE Healthcare, Piscataway, NJ, USA) and a buffer containing 20 mM Tris-HCl, pH 8.0; 150 mM NaCl; and 2 mM DTT. The fractions containing PLpro were pooled; exchanged into 20 mM Tris-HCl, pH 8.0, and 10 mM DTT; and concentrated to 10 mg/mL by ultrafiltration (Microcon YM-30, Millipore Corporation, Bedford, MA, USA). The protein purity was examined by conducting SDS-PAGE and native PAGE. The protein concentration was determined using the Bradford method [25] with bovine serum albumin used as the standard. The N-terminal his tag was removed by TEV digestion for the activity assay.

### 4.5. SARS-CoV PLpro Activity Inhibition Assay

IC_50_ values for all inhibitors were determined using a 396-well plate-based assay, similar to our previously reported procedures [26,27]. Fluorogenic peptide Z-Arg-Leu-Arg-Gly-Gly-AMC (Z-RLRGG-AMC), purchased from ENZO Life Sciences, was used as the substrate. The substrate contains five C-terminal residues of human ubiquitin with a C-terminal 7-amido-4-methylcoumarin (AMC) group. Hydrolysis of the AMC–peptide bond dramatically increases the fluorescence, allowing conversion to be accurately determined. Reactions were performed in a total volume of 50 μL, containing the following components: 20 mM Tris buffer, pH 8.0; 10 mM DTT; 250 μM Z-RLRGG-AMC; 2% DMSO; and varying concentrations of the inhibitor (0–200 μM). Reactions were initiated with the addition of PLpro to produce a final enzyme concentration of 142 nM. The reaction progress was continuously monitored on a SpectraMax M3Multi-Mode Microplate Reader (λ_excitation_ = 360 nm; λ_emission_ = 460 nm; gain = 40). The initial rate data were fit to the equation *vi* = *vo*/(1 + [*I*]/IC_50_) using the enzyme kinetics module of Sigma Plot (v. 9.01 Systat Software, Inc.), where *vi* is the reaction rate in the presence of the inhibitor, *vo* is the reaction rate in the absence of the inhibitor, and [*I*] is the inhibitor concentration.

### 4.6. Statistical Analyses

All measurements were performed in triplicate. The results were subjected to a variance analysis using a Sigma plot. Differences were considered significant at *p* < 0.05.

## Figures and Tables

**Table 1:** In vitro SARS-CoV PL^pro^ inhibitory activity of pterocarpane derivatives (**1**–**6**), chalcones (**7**, **8**), and isoflavanone (**9**).

Inhibitor	IC_50_ ^a^ Value (μM)	Inhibition Mode (*K*_i_, μM) ^b^
Erythrabyssin II (**1**)	18.2 ± 2.3	Noncompetitive (17.0 ± 3.1)
Lespebuergine G4 (**2**)	32.9 ± 1.4	Noncompetitive (33.2 ± 1.7)
1-Methoxyerythrabyssin II (**3**)	11.2 ± 0.9	Noncompetitive (8.6 ± 1.1)
Bicolosin A (**4**)	43.2 ± 1.8	Noncompetitive (45.2 ± 3.1)
Bicolosin B (**5**)	217.4 ± 7.6	NT ^c^
Bicolosin C (**6**)	9.3 ± 3.1	Noncompetitive (9.0 ± 1.4)
Xanthoangelol (**7**)	6.3 ± 1.5	Noncompetitive (5.5 ± 0.8)
(±)-Lespeol (**8**)	50.1 ± 4.2	Noncompetitive (54.0 ± 4.5)
Parvisoflavanone (**9**)	103.8 ± 6.1	Noncompetitive (75.3 ± 5.7)

^a^ All compounds were examined in a set of experiments repeated three times; IC_50_ values of compounds represent the concentration that caused 50% enzyme activity loss. ^b^ Values of the inhibition constant. ^c^ NT means not tested.

## Data Availability

All available data are reported in the paper.

## Supplementary Materials

The following supporting information can be downloaded at: https://www.mdpi.com/article/10.3390/plants13233319/s1, Figure S1: 1H-NMR spectrums of compound **1**, Figure S2: 13C-NMR spectrums of compound **1**, Figure S3: EIMS and HREIMS data of compound **1**, Figure S4: 1H-NMR spectrums of compound **2**, Figure S5: 13C-NMR spectrums of compound **2**, Figure S6: EIMS and HREIMS data of compound **2**, Figure S7: 1H-NMR spectrums of compound **3**, Figure S8: 13C-NMR spectrums of compound **3**, Figure S9: EIMS and HREIMS data of compound **3**, Figure S10: 1H-NMR spectrums of compound **4**, Figure S11: 13C-NMR spectrums of compound **4**, Figure S12: EIMS and HREIMS data of compound **4**, Figure S13: 1H-NMR spectrums of compound **5**, Figure S14: 13C-NMR spectrums of compound **5**, Figure S15: EIMS and HREIMS data of compound **5**, Figure S16: 1H-NMR spectrums of compound **6**, Figure S17: 13C-NMR spectrums of compound **6**, Figure S18: EIMS and HREIMS data of compound **6**, Figure S19: 1H-NMR spectrums of compound **7**, Figure S20: 13C-NMR spectrums of compound **7**, Figure S21: EIMS and HREIMS data of compound **7**, Figure S22: 1H-NMR spectrums of compound **8**, Figure S23: 13C-NMR spectrums of compound **8**, Figure S24: EIMS and HREIMS data of compound **8**, Figure S25: 1H-NMR spectrums of compound **9**, Figure S26: 13C-NMR spec-trums of compound **9**, Figure S27: EIMS and HREIMS data of compound **9**, Figure S28: CD spectra of 1-6, Figure S29: Lineweaver-Burk Plot for the inhibition of compounds, Figure S30: Dixon Plot for the inhibition of compounds, Figure S31: SARS-CoV PLpro purified from *E. Coli*, Figure S32: SARS-CoV PLpro purified from *E. Coli*.
