# Supplementary material for: Flavonoids Derived from the Roots of Lespedeza bicolor Inhibit the Activity of SARS-CoV Papain-like Protease
Source: Plants (Basel). 2024 Nov 26;13(23):3319. doi: 10.3390/plants13233319 (PMC11644518; doi:10.3390/plants13233319)
Supplement: Supplementary file 1 [file plants-13-03319-s001.zip › plants-3301192-supplementary 2.pdf]

▣ Characterization Data

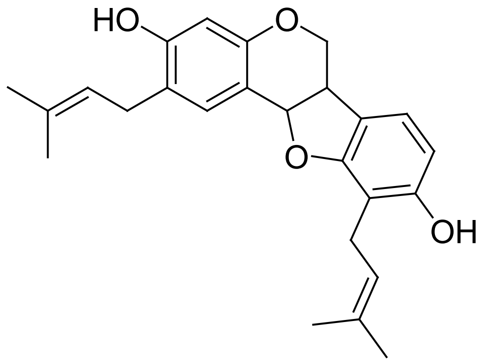

Colourless needless; mp 161–163 °C; mp 161–163 °C; $[\alpha]^{20}_{D}$ -104 (*c* 0.25 MeOH); IR (KBr) cm^{-1} 3434, 2923, 2858, 1621, 1449, 1379, 1116, 1033, 607; CD (MeOH) λ_{max} nm 288 (4.532); UV (MeOH) λ_{max} (log ε) 287 (1.702) nm; ^{1}H NMR (500 MHz, $CDCl_3$) δ 1.66 (3H, s, H-5''), 1.71 (6H, s, H-5', 4''), 1.76 (3H, s, H-4'), 3.27 (2H, m, H-1'), 3.32 (2H, m, H-1''), 3.42 (1H, m, H-6a), 3.51 (1H, t, *J* = 11.0 Hz, H-6), 4.12 (1H, dd, *J* = 5.0 Hz, 10.0 H-6), 5.21 (2H, t, *J* = 5.0 Hz, H-2''), 5.26 (1H, d, *J* = 5.0 Hz, H-2'), 5.36 (1H, d, *J* = 5.0 Hz, H-11a), 6.26 (1H, d, *J* = 5.0 Hz, H-8), 6.32 (1H, s, H-4), 6.88 (1H, d, *J* = 1.6 Hz, H-7), 7.17 (1H, s, H-1).

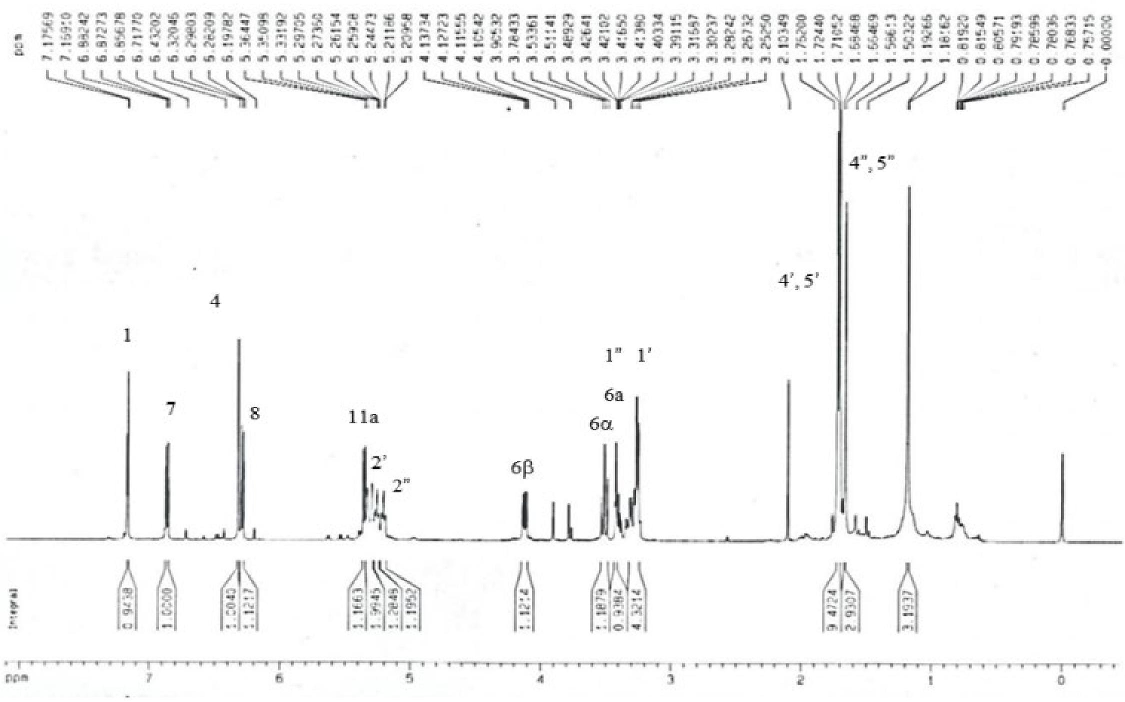

Figure S1: ^{1}H- NMR spectrums of compound **1**

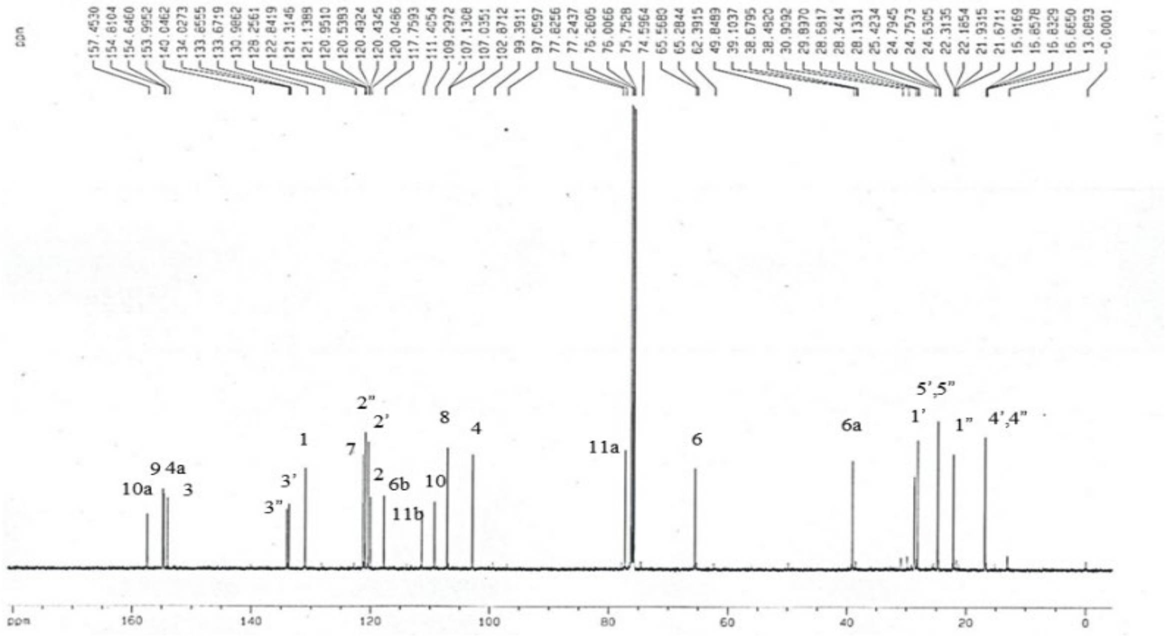

Figure S2: ^{13}C-NMR spectrums of compound **1**

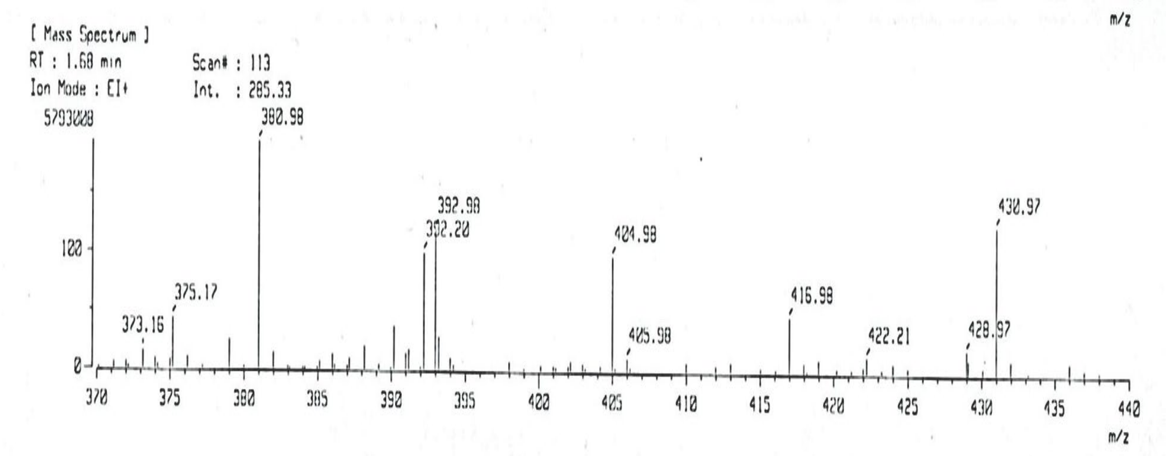

[Theoretical Ion Distribution]
Molecular Formula : C25 H28 O4
(m/z 392.1988, MW 392.4949, U.S. 12.0)
Base Peak : 392.1988, Averaged MW : 392.4965(a), 392.4973(w)

Observed m/z	Int%	Err[ppm / mmu]	U.S.	Composition
392.1989	100.0	+0.3 / +0.1	12.0	C 25 H 28 O 4

Figure S3: EIMS and HREIMS data of compound **1**

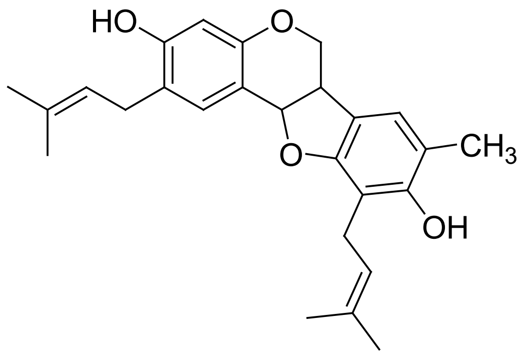

Colorless amorphus solid; mp 161–163 °C ; $[\alpha]_D^{20}$ -199 (c 0.20 MeOH); IR (KBr) cm^{-1} 3442, 2924, 2854, 1629, 1458, 1120, 1021, 580; CD (MeOH) λ_{max} nm ([θ]) 291 (36.239); UV (MeOH) $\lambda_{max}(\log \varepsilon)$ 291 (1.849) nm; ^{1}H NMR (500 MHz, $CDCl_3$) δ 1.81 (3H, s, H-5''), 1.84 (6H, s, H-4', 5'), 1.88 (3H, s, H-4''), 2.22 (3H, s, CH3), 3.38 (2H, d, J = 7.2 Hz, H-1'), 3.43 (1H, m, H-1''), 3.50 (1H, m, H-6a), 3.65 (1H, t, J = 11.1 Hz, H-6), 4.25 (1H, dd, J = 5.1, 10.9 Hz, H-6), 5.34 (1H, t, J = 5.8 Hz, H-2''), 5.40 (1H, t, J = 6.1 Hz, H-2'), 5.45 (1H, d, J = 6.6 Hz, H-11a), 6.44 (1H, s, H-4), 6.91 (1H, s, H-7), 7.29 (1H, s, H-1).

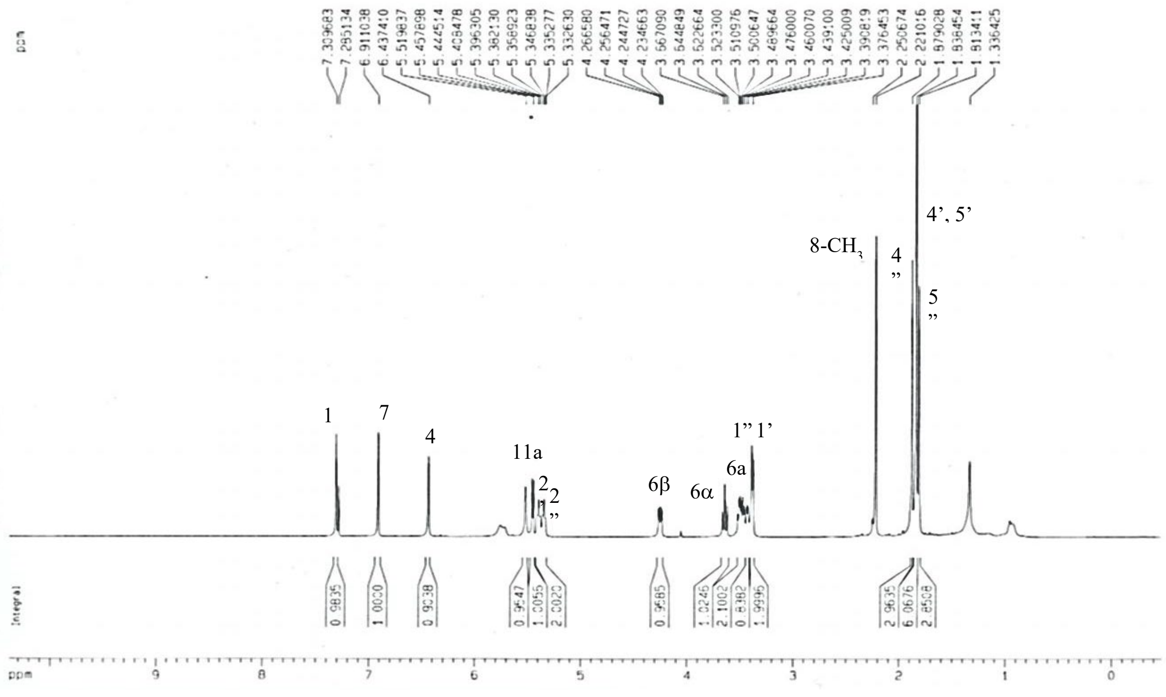

Figure S4: ^{1}H- NMR spectrums of compound 2

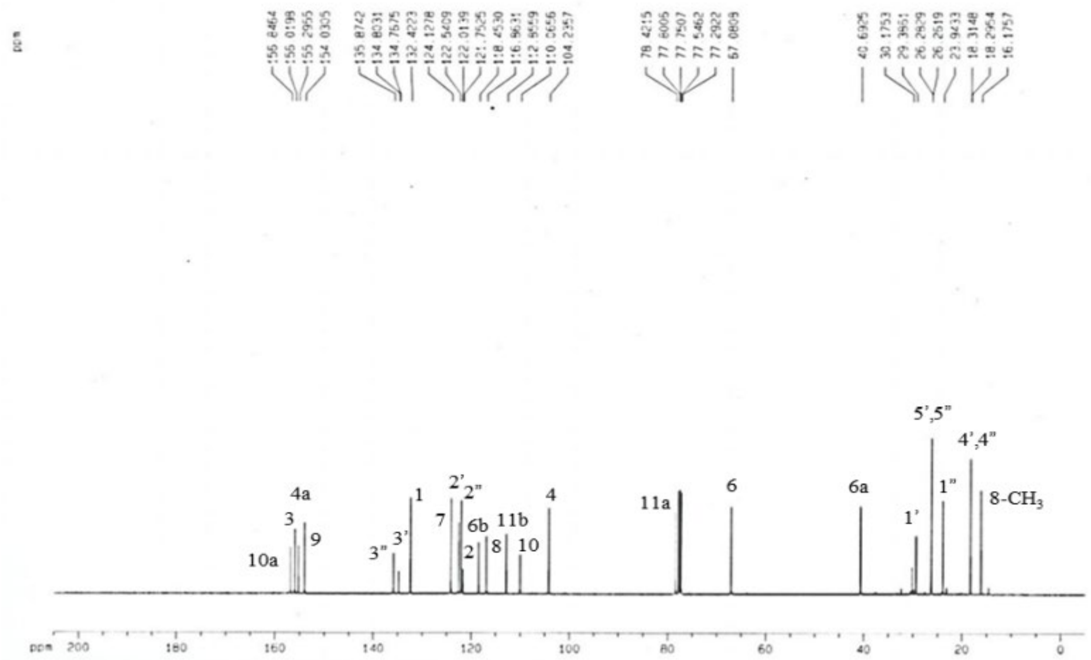

Figure S5: ^{13}C-NMR spectrums of compound 2

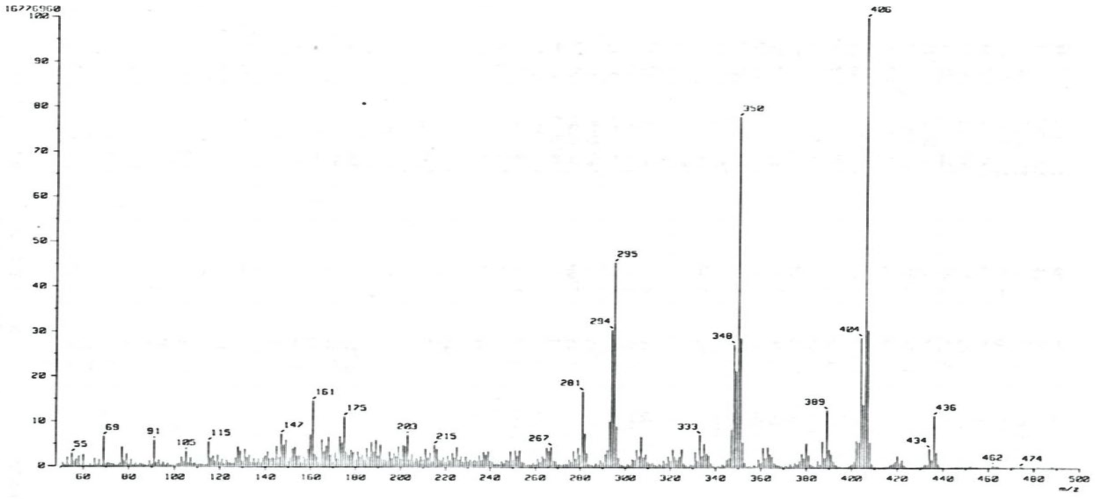

[Elemental Composition]
Data : WSL-10 Date : 03-May-2010 15:46
Sample: -
Note : -
Inlet : Direct Ion Mode : EI+
RT : 0.54 min Scan#: 17
Elements : C 100/1, H 100/1, O 20/1
Mass Tolerance : 1000ppm, 10mmu if m/z > 10
Unsaturation (U.S.) : 0.0 - 20.0

Observed m/z	Int%	Err[ppm / mmu]	U.S.	Composition
406.2141	100.0	-0.7 / -0.3	12.0	C 26 H 30 O 4
		-15.2 / -6.2	3.0	C 19 H 34 O 9

Figure S6: EIMS and HREIMS data of compound **2**

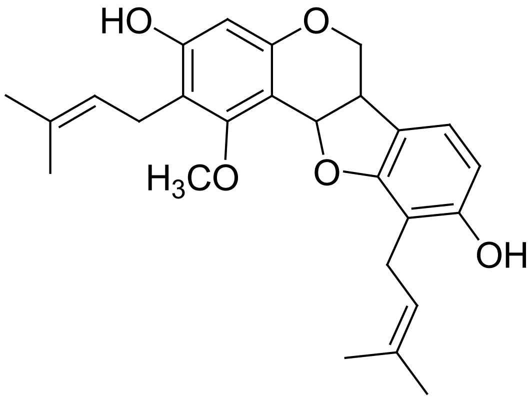

Pale yellow; mp 161–163 °C ; $[\alpha]_D^{20}$ -145 (c 0.24 MeOH); IR (KBr) cm^{-1} 3438, 2921, 2851, 1618, 1498, 1458, 1383, 1271, 1211, 1129, 1101, 958, 760, 585; CD (MeOH) λ_{max} nm ([θ]) 285 (4.4363); UV (MeOH) λ_{max} (log ε) 291 (1.625) nm; ^{1}H NMR (500 MHz, $CDCl_3$) δ 1.76 (3H, s, H-5'), 1.78 (3H, s, H-5"), 1.80 (3H, s, H-4'), 1.87 (3H, s, H-4"), 3.36 (2H, brm, H-1"), 3.39 (1H, brm, H-6a), 3.48 (2H, brm, H-1'), 3.63 (1H, t, *J* = 11.2 Hz, H-6), 4.05 (3H, s, OCH_3), 4.19 (1H, dd, *J* = 5.1, 10.9 Hz, H-6), 5.30 (1H, brt, H-2'), 5.36 (1H, brt, H-2"), 5.66 (1H, d, *J* = 6.4 Hz, H-11a), 6.31 (1H, s, H-4), 6.39 (1H, d, *J* = 7.9 Hz, H-8), 6.96 (1H, d, *J* = 7.9 Hz, H-7).

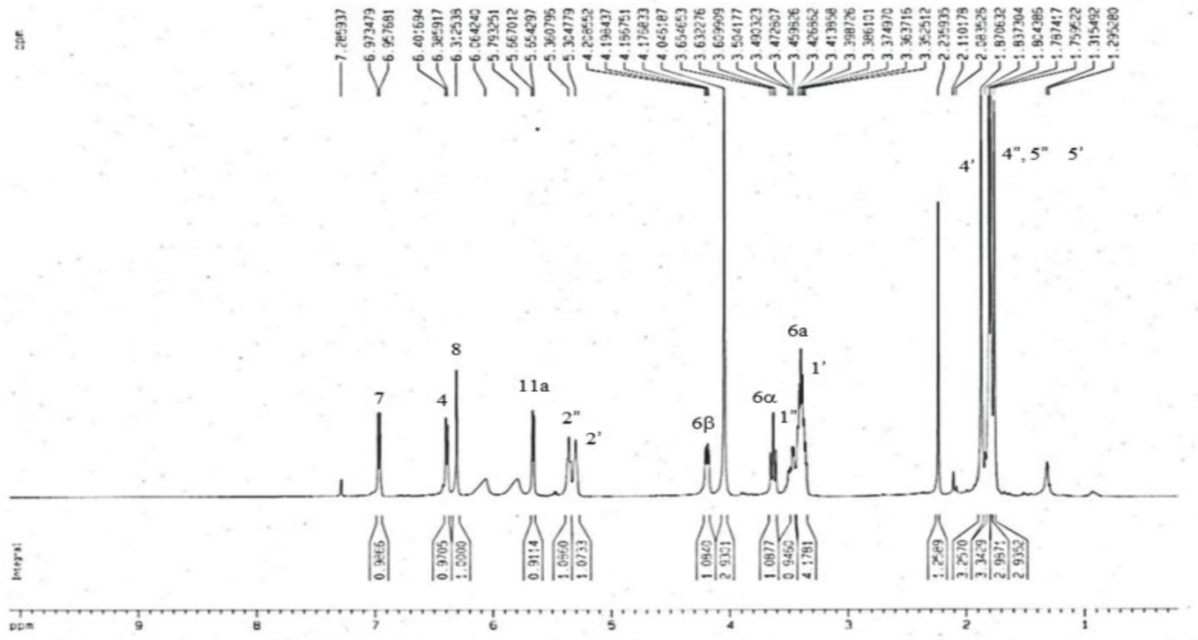

Figure S7: ^{1}H-NMR spectrums of compound **3**

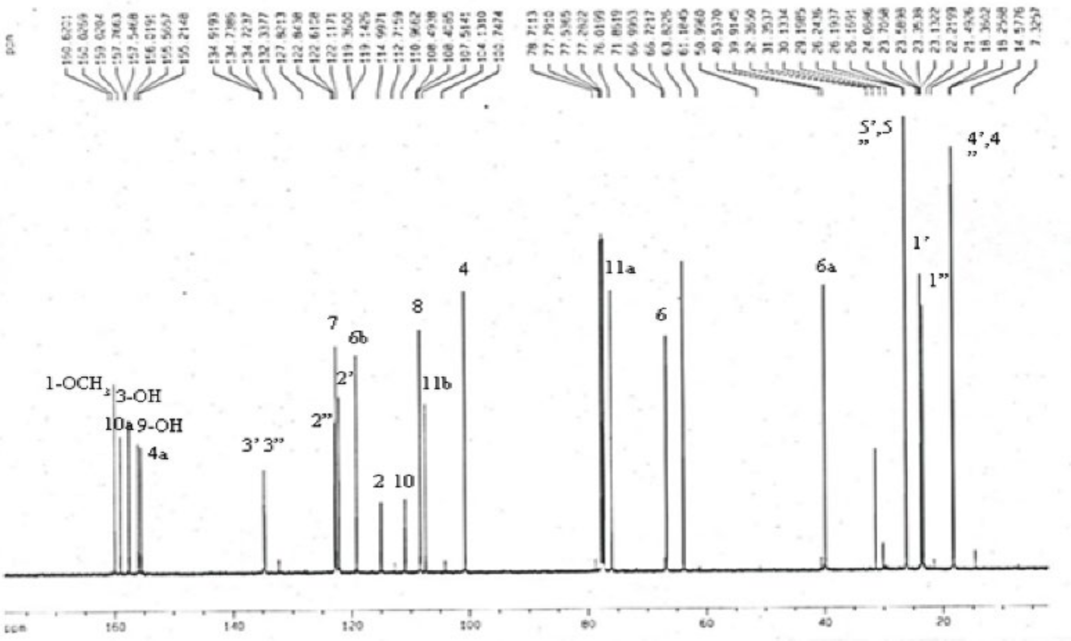

Figure S8: ^{13}C-NMR spectrums of compound **3**

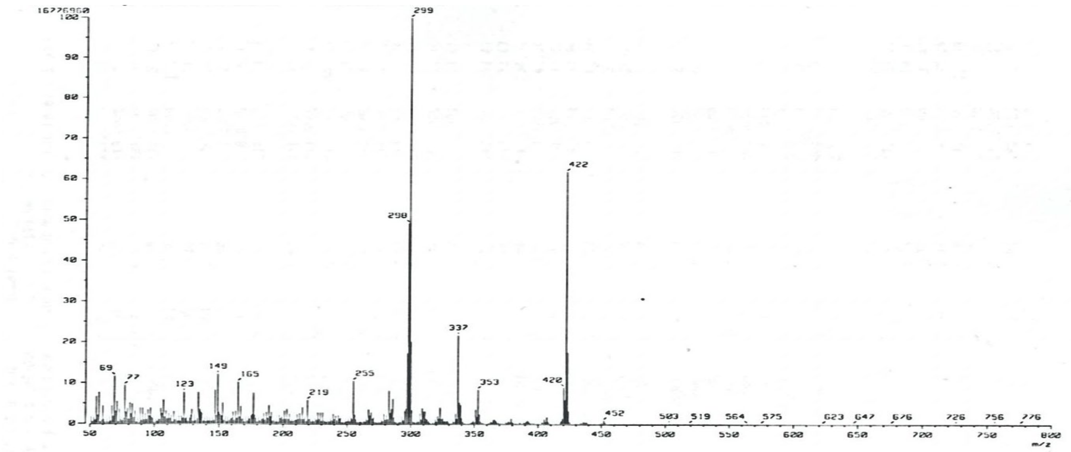


```
[ Elemental Composition ]
Data   : WSL52                               Date : 14-Sep-2010 16:43
Sample: -
Note : -
Inlet : Direct                               Ion Mode : EI+
RT : 1.44 min                                Scan#: 44
Elements : C 100/1, H 100/1, O 20/1
Mass Tolerance         : 1000ppm, 1mmu if m/z < 1, 2mmu if m/z > 2
Unsaturation (U.S.)  : 1.0 - 30.0

Observed m/z  Int%    Err[ppm / mmu]     U.S.   Composition
  422.2096    100.0      +0.7 /  +0.3    12.0   C 26 H 30 O 5
```

Figure S9: EIMS and HREIMS data of compound **3**.

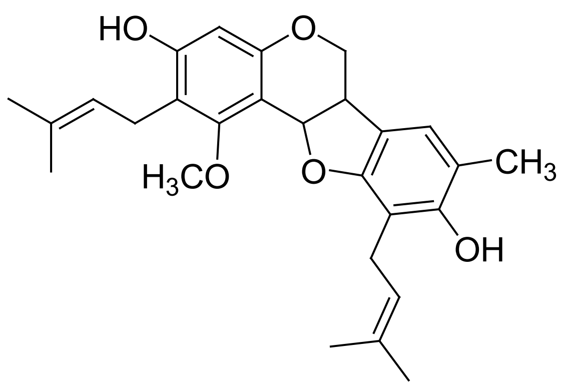

White powder; mp 161–163 °C; $[\alpha]_D^{20}$ -71 (*c* 0.23 MeOH); IR (KBr) cm^{-1} 3433, 2978, 2917, 1617, 1436, 1040, 956, 703, 577; CD (MeOH) λ_{max} nm ([θ]) 285 (4.64); UV (MeOH) λ_{max} (log ε) 286 (1.757) nm; ^{1}H NMR (500 MHz, $CDCl_3$) δ 1.67 (3H, s, H-5'), 1.68 (3H, s, H-5''), 1.74 (6H, s, H-4'), 1.76 (3H, s, H-4''), 2.09 (3H, s, CH3), 3.26 (1H, m, H-6a), 3.30 (1H, m, H-1'), 3.34 (1H, m, H-1''), 3.51 (1H, t, *J* = 11.1 Hz, H-6), 3.90 (3H, s, OCH3), 4.09 (1H, dd, *J* = 5.1, 11.0 Hz, H-6), 5.17 (1H, t, *J* = 6.7 Hz, H-2'), 5.23 (1H, t, *J* = 7.4 Hz, H-2''), 5.30 (OH, s), 5.48 (OH, s), 5.50 (1H, d, *J* = 6.4 Hz, H-11a), 6.20 (1H, s, H-4), 6.79 (1H, s, H-7).

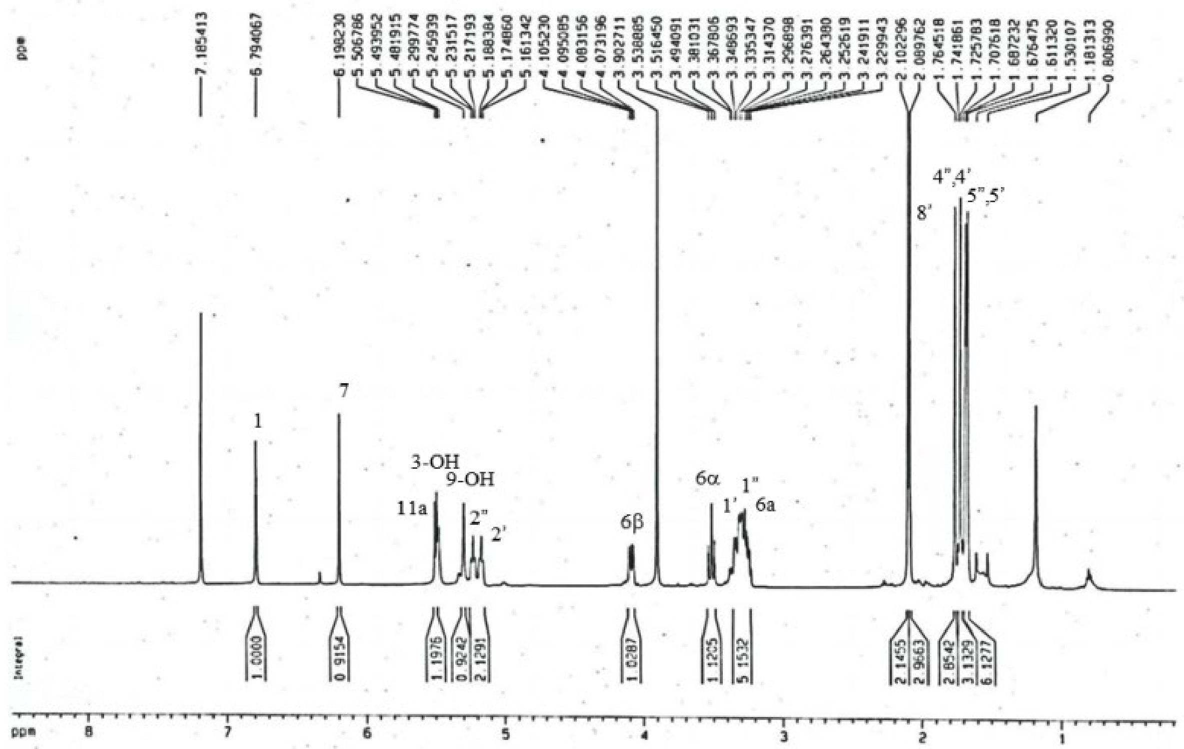

Figure S10: ^{1}H-NMR spectrums of compound **4.**

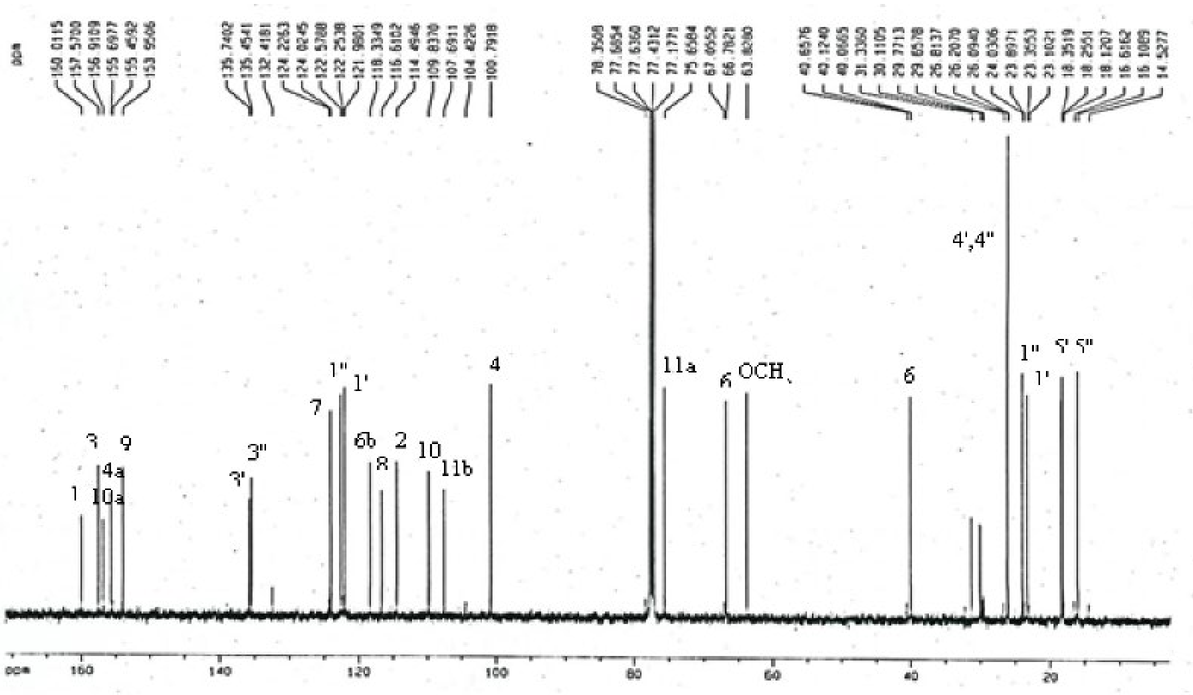

Figure S11: ^{13}C-NMR spectrums of compound **4.**

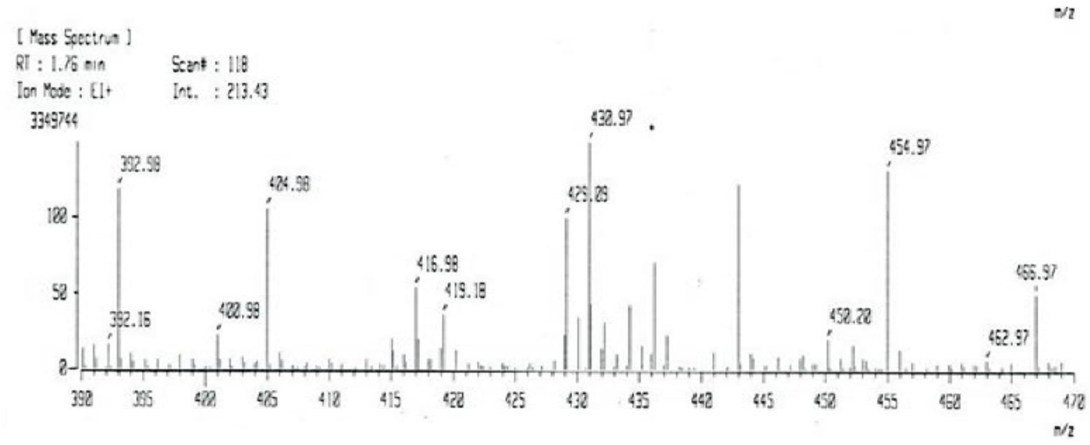

[Theoretical Ion Distribution]
Molecular Formula : C27 H32 O5
(m/z 436.2250, MW 436.5481, U.S. 12.0)
Base Peak : 436.2250, Averaged MW : 436.5498(a), 436.5505(w)

Observed m/z	Int%	Err[ppm / mmu]	U.S.	Composition
436.2250	71.0	+0.1 / +0.1	12.0	C 27 H 32 O 5

Figure S12: EIMS and HREIMS data of compound **4**.

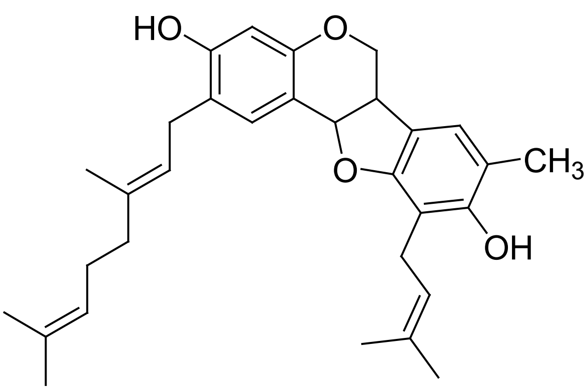

Yellow powder; mp 161–163 °C; $[\alpha]_D^{20}$ -149 (*c* 0.21 MeOH); IR (KBr) cm^{-1} 3433, 2924, 2853, 1637, 1495, 1469, 1384, 1285, 1161, 1071, 1023, 958, 830, 535; CD (MeOH) λ_{max} nm ([θ]) 291 (26.58); UV (MeOH) λ_{max} (log ε) 291 (1.698) nm; ^{1}H NMR (500 MHz, $CDCl_3$) δ 1.53 (3H, s, H-8'), 1.61 (3H, s, H-9'), 1.67 (3H, s,H-5"), 1.71 (3H, s, H-10'), 1.74 (3H, s, H-4"), 2.02 (3H, brm, H-4'), 2.04 (3H, brm, H-5'), 2.08 (3H, s, CH_3), 3.28 (2H, m, H-1'), 3.37 (1H, m, H-1"), 3.38 (2H, m, H-6a), 3.51 (1H, t, *J* = 10.8 Hz, H-6), 4.12 (1H, dd, *J* = 5.0, 10.9 Hz, H-6), 5.0 (1H, t, *J* = 6.5 Hz, H-6'), 5.2 (1H, t, *J* = 7.2 Hz, H-2"), 5.26 (1H, t, *J* = 6.0 Hz, H-2'), 5.31 (1H, d, *J* = 6.7 Hz, H-11a), 5.34 (9-OH, s) 6.33 (1H, s, H-4), 6.78 (1H, s, H-7), 7.18 (1H, s, H-1).

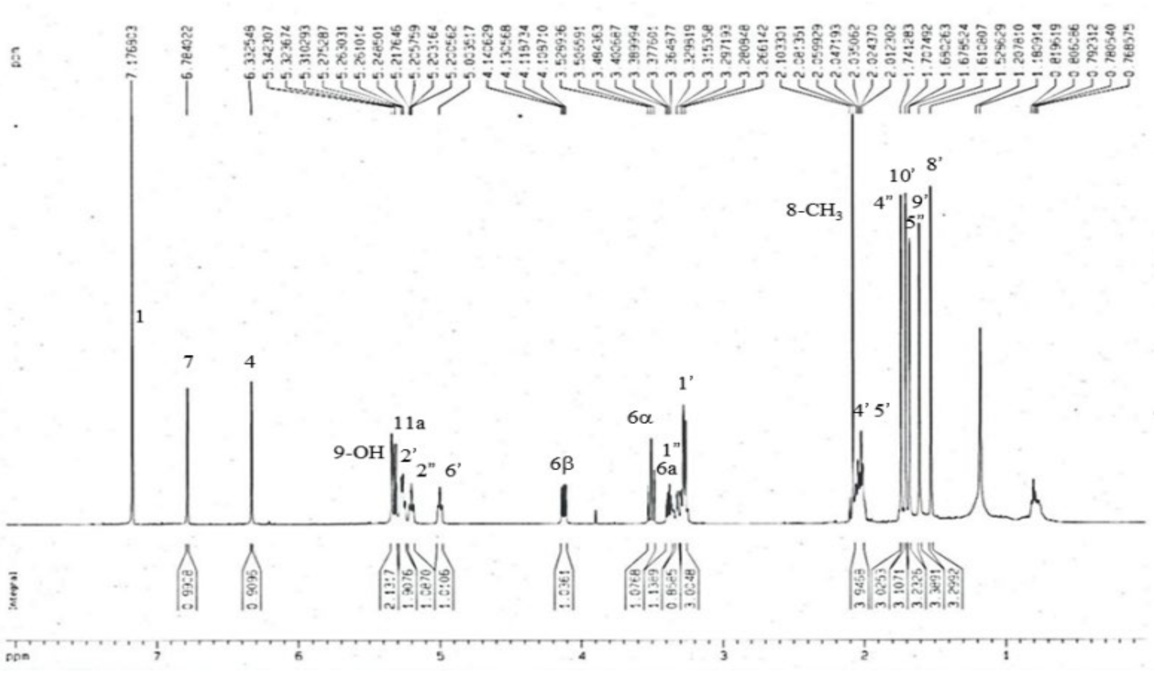

Figure S13: ^{1}H-NMR spectrums of compound **5.**

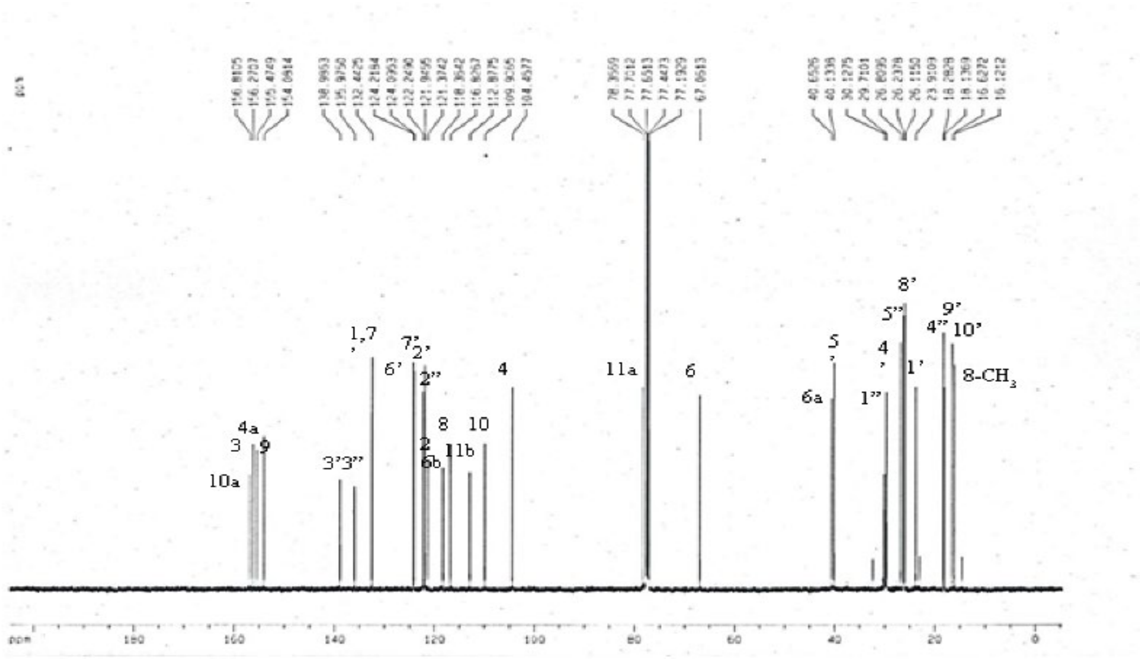

Figure S14: ^{13}C-NMR spectrums of compound **5.**

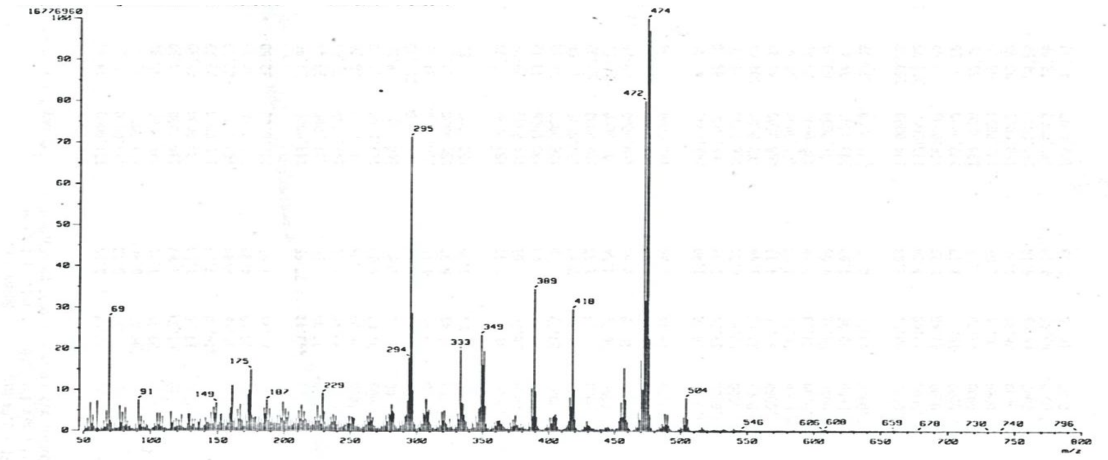


```
[ Mass Spectrum ]
Data   : WSL9                                   Date : 01-Jul-2010 16:22
Sample: -
Note  : -
Inlet : Direct                                  Ion Mode : EI+
Spectrum Type : Normal Ion [MF-Linear]
RT : 2.04 min            Scan# : 62
BP : m/z 474.0000        Int.   : 1599.98
Output m/z range : 50.0000 to 800.0000          Cut Level : 1.00 %
```

Observed m/z	Int%	Err[ppm / mmu]	U.S.	Composition
474.2773	100.0	+0.6 / +0.3	13.0	C 31 H 38 O 4

Figure S15: EIMS and HREIMS data of compound **5**.

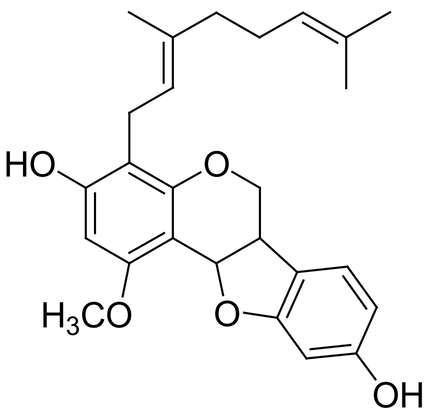

Yellow amorphous plate; mp 161–163 °C ; $[\alpha]_D^{20}$ -122 (*c* 0.21 MeOH); IR (KBr) cm^{-1} 3428, 2921, 2851, 1700, 1622, 1512, 1454, 1383, 1270, 1161, 1112, 1026, 975, 830, 838, 538, 413; CD (MeOH) λ_{max} nm ([θ]) 286 (20.802); UV (MeOH) λ_{max} (log ε) 283 (1.782) nm; ^{1}H NMR (500 MHz, $CDCl_3$) δ 1.50 (3H, s, H-8'), 1.59 (3H, s, H-9'), 1.70 (3H, s, H-10'), 1.95 (2H, brt, *J* = 12.6 Hz, H-4'), 2.00 (2H, brt, *J* = 13.2 Hz, H-5'), 3.25 (2H, brm, H-1'), 3.27 (2H, brm, H-6a), 3.49 (1H, t, *J* = 10.3 Hz, H-6), 3.70 (3H, s, OCH3), 4.13 (1H, dd, *J* = 5.6, 11.3 Hz, H-6), 4.97 (1H, t, *J* = 6.6 Hz, H-6'), 5.14 (1H, t, *J* = 6.6 Hz, H-2'), 5.57 (1H, d, *J* = 12.2 Hz, H-11a), 6.03 (1H, s, H-2), 6.26 (1H, d, *J* = 13.7 Hz, H-8), 6.30 (1H, s, H-10), 6.95 (1H, d, *J* = 14.4 Hz, H-7).

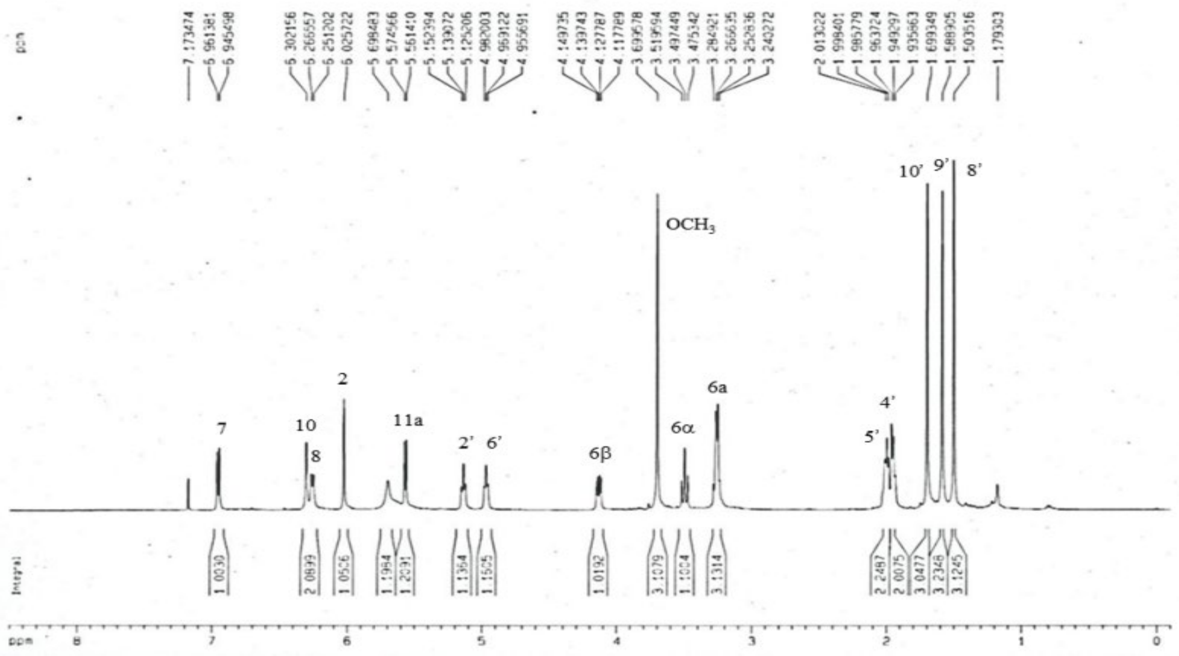

Figure S16: ^{1}H-NMR spectrums of compound **6**

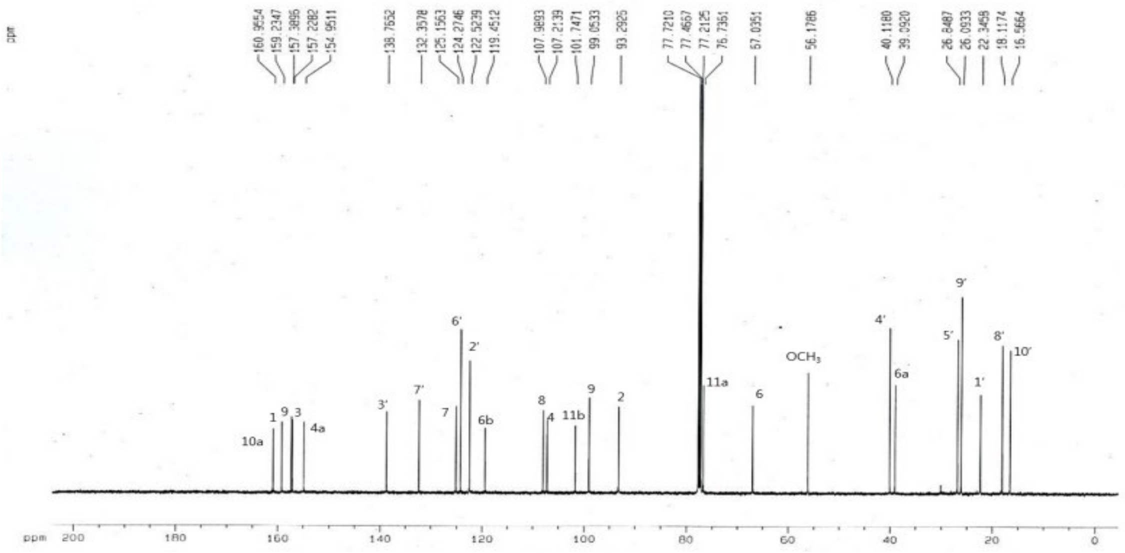

Figure S17: ^{13}C-NMR spectrums of compound **6**

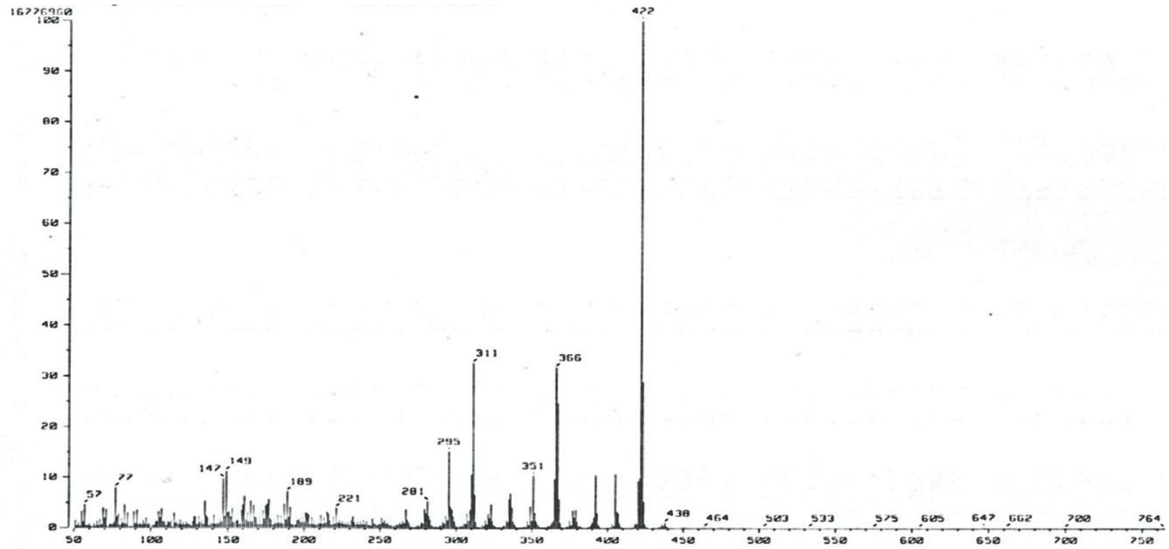


```
[ Elemental Composition ]
Data   : WSL32                                Date : 14-Sep-2010 16:52
Sample: -
Note : -
Inlet : Direct                                Ion Mode : EI+
RT : 0.80 min                                 Scan#: 25
Elements : C 100/1, H 100/1, O 20/1
Mass Tolerance       : 1000ppm, 1mmu if m/z < 1, 2mmu if m/z > 2
Unsaturation (U.S.) : 1.0 - 30.0

Observed m/z Int%   Err[ppm / mmu]     U.S.  Composition
  422.2092   100.0     -0.4 /  -0.2    12.0  C 26 H 30 O 5
```

Figure S18: EIMS and HREIMS data of compound **6**

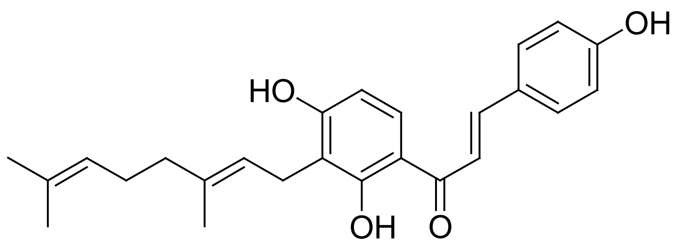

Yellow gum; UV (MeOH) λ_{max} (log ε) 224 (sh) (4.70), 280, (4.45) nm; ^{1}H NMR (500 MHz, $CDCl_3$) δ 1.59 (3H, s, H-9"), 1.67 (3H, s, H-8"), 1.83 (3H, s, H-10"), 2.10 (4H, brm, H-4" and H-5"), 3.49 (2H, d, J = 7.3 Hz, H-1"), 5.05 (1H, brt, J = 6.4 Hz, H-6"), 5.30 (1H, t, J = 6.9 Hz, H-2"), 6.42 (1H, d, J = 8.7 Hz, h-5'), 6.88 (2H, d, J = 6.85 Hz, H-3 and 5), 7.46 (1H, d, H = 10.3 Hz, H-α), 7.55 (2H, d, J = 10.3 Hz, H-2 and 6), 7.71 (1H, d, J = 8.8 Hz, H-6'), 7.84 (1H, d, J = 15.3 Hz, H-β), 13.88 (1H, s, 2'-OH).

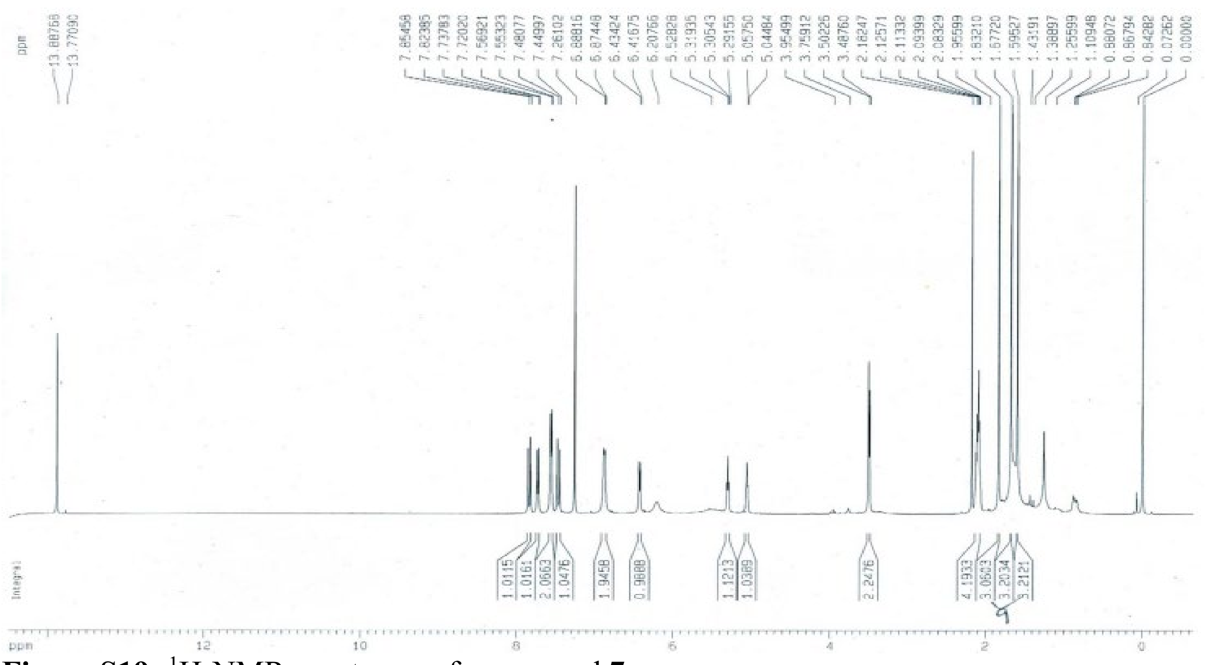

Figure S19: ^{1}H-NMR spectrums of compound **7**

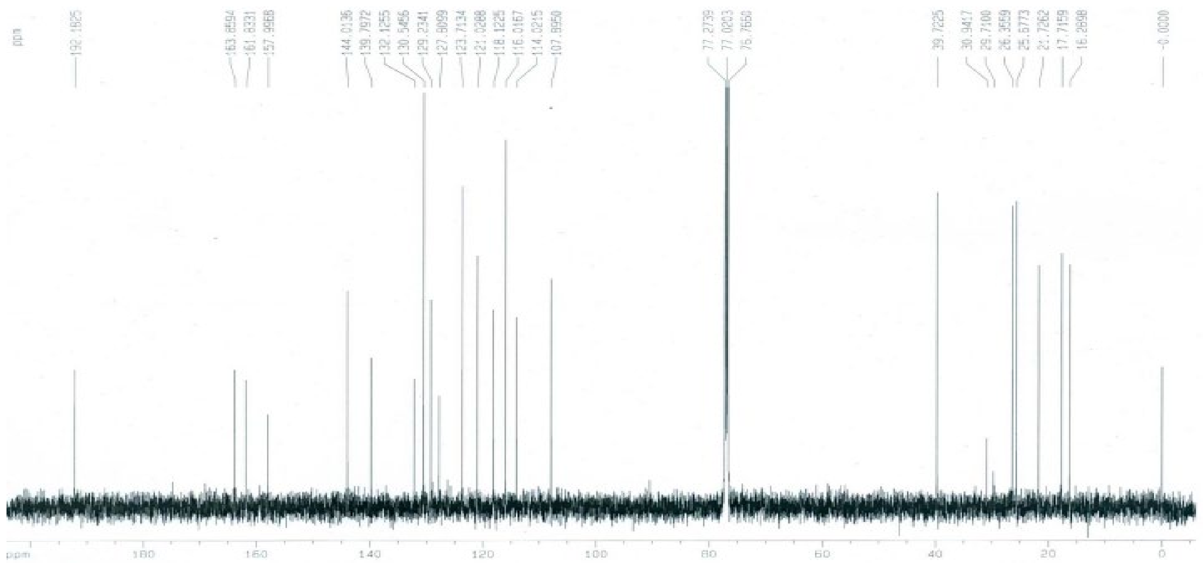

Figure S20: ^{13}C-NMR spectrums of compound **7**

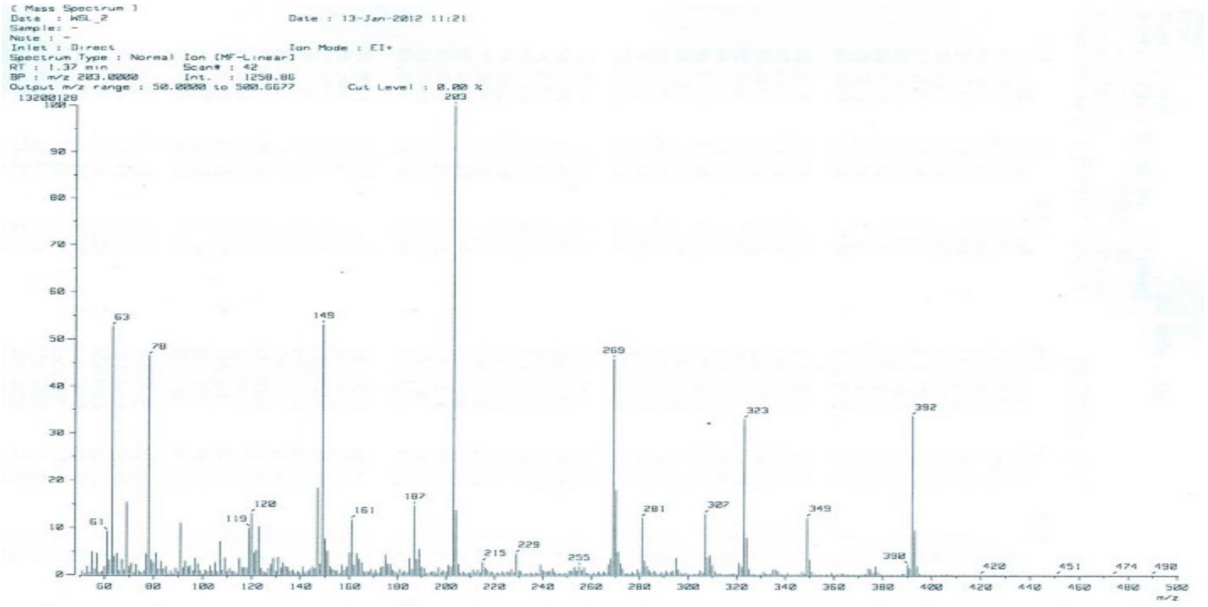

[Elemental Composition]　　Page: 1

Data : WSL_2　　Date : 13-Jan-2012 14:32

Sample: -

Note : -

Inlet : Direct　　Ion Mode : EI+

RT : 1.35 min　　Scan#: 28

Elements : C 100/1, H 100/1, O 10/1

Mass Tolerance : 3mmu

Unsaturation (U.S.) : 0.0 - 30.0

Observed m/z	Int%	Err[ppm / mmu]	U.S.	Composition
392.1986	100.0	-0.4 / -0.2	12.0	C 25 H 28 O 4

Figure S21: EIMS and HREIMS data of compound **7**

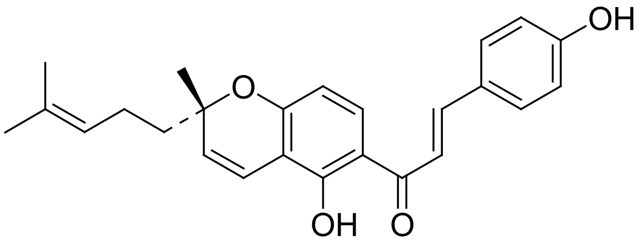

Orange-yellow gum; [a]D + 21o ($CDCl_3$, c 0.12); UV (MeOH) λ_{max} (log ε) 226 (4.39), 276, (4.35), 370 (4.19) nm, ^{1}H NMR (500 MHz, $CDCl_3$) δ 1.36 (3H, s, H-10''), 1.50 (3H, s, H-9''), 1.58 (3H, s, H-8''), 1.61 (1H, m, Ha-5''), 1.69 (1H, m, Hb-5''), 2.02 (2H, m, H-4''), 5.03 (1H, m, H-6''), 5.46 (1H, d, *J* = 10.1 Hz, H-2''), 6.29 (1H, d, J = 8.9 Hz, H-1''), 6.29 (1H, d, *J* = 8.9 Hz, H-5'), 6.72 (2H, d, *J* = 10.1 Hz, H-3 and 5), 7.35 (1H, d, *J* = 15.4 Hz, H-a), 7.49 (2H, d, *J* = 8.6 Hz, H-2 and H-6), 7.63 (1H, d, *J* = 8.9 Hz, H-6), 7.75 (1H, d, *J* = 15.4 Hz, H-β)

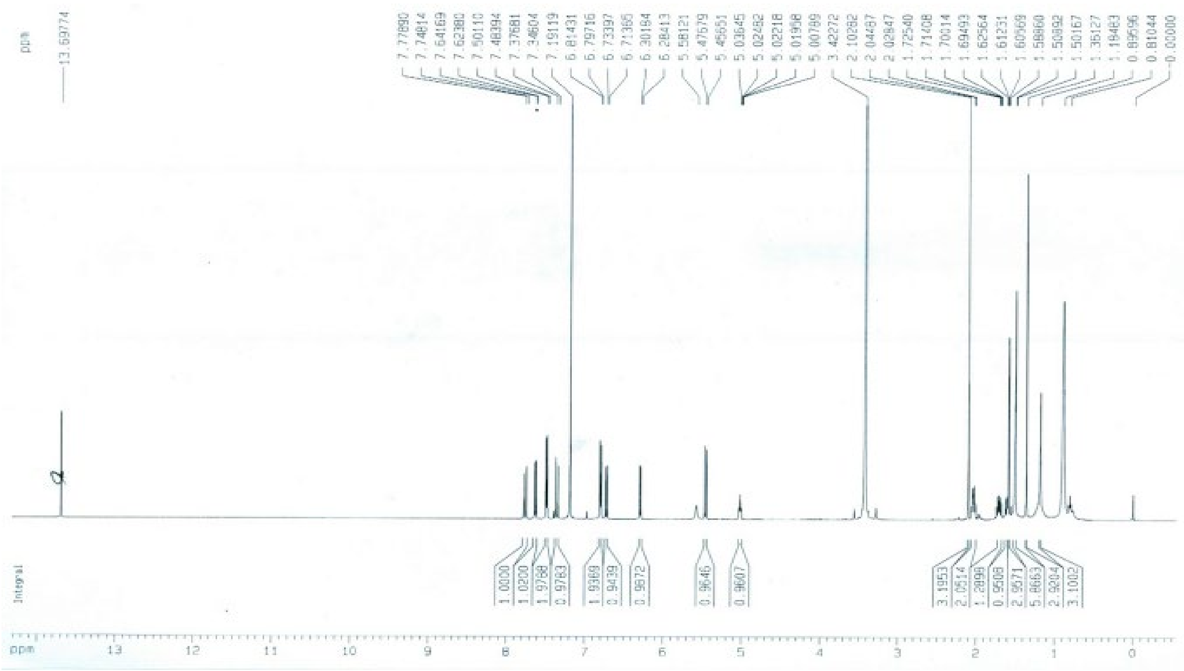

Figure S22: ^{1}H-NMR spectrums of compound **8**

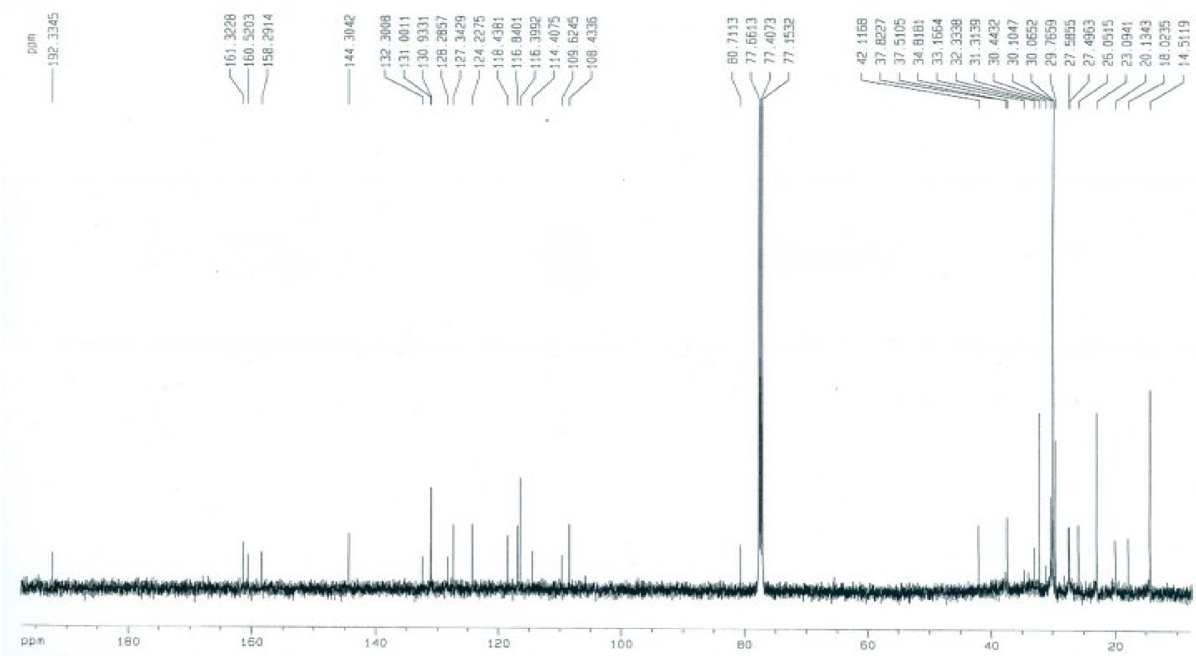

Figure S23: ^{13}C-NMR spectrums of compound **8**

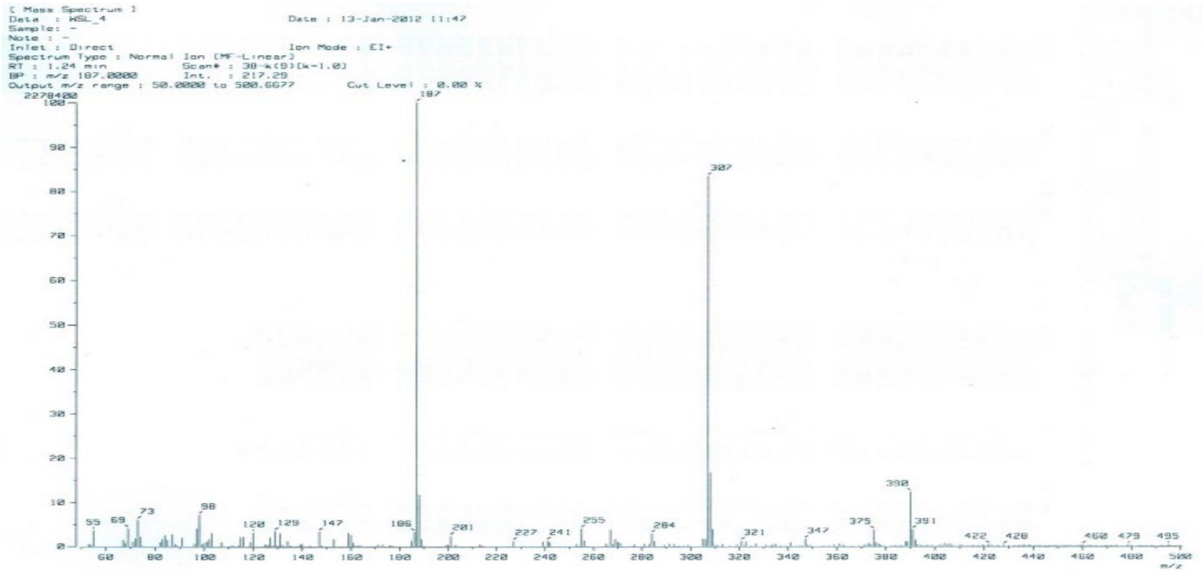

[Elemental Composition]   Page: 1
Data : WSL_4   Date : 13-Jan-2012 14:23
Sample: -
Note : -
Inlet : Direct   Ion Mode : EI+
RT : 1.20 min   Scan#: 25
Elements : C 100/1, H 100/1, O 10/1
Mass Tolerance : 3mmu
Unsaturation (U.S.) : 0.0 - 30.0

Observed m/z	Int%	Err[ppm / mmu]	U.S.	Composition
390.1835	50.7	+0.9 / +0.4	13.0	C 25 H 26 O 4

Figure S24: EIMS and HREIMS data of compound **8**

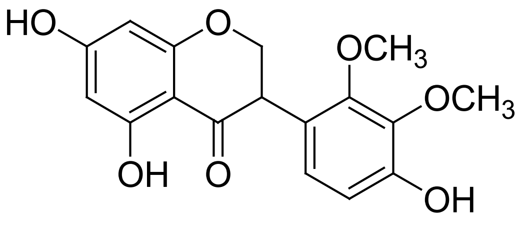

Colourless prisms, m.p. 201-203ºC ^{1}H NMR (300 MHz, CDCl3) δ 3.86 (s, OCH3), 3.89 (s, OCH3), 4.25 (m, H-2b), 4.45 (m, H-2a), 4.52 (m, H-3), 5.96 (s, H-8), 6.01 (s, H-6), 6.71 (d, J = 8.6 Hz, H-6'), 6.78 (d, J = 8.6 Hz, H-5'), 12.26 (s, 5-OH)

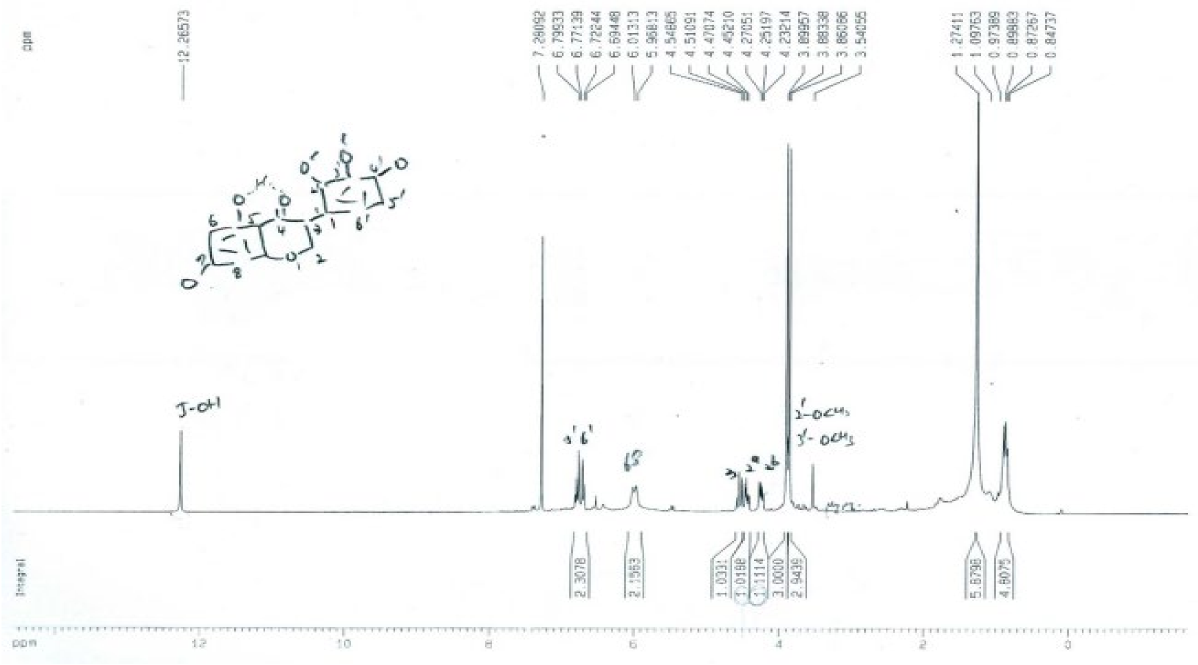

Figure S25: ^{1}H-NMR spectrums of compound **9**

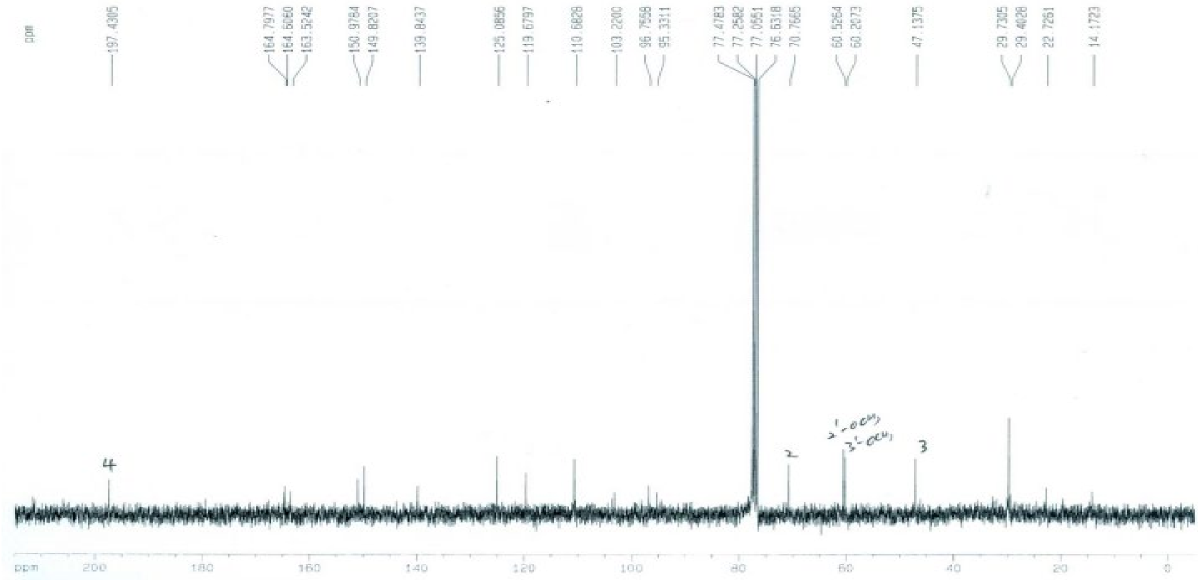

Figure S26: ^{13}C-NMR spectrums of compound **9**

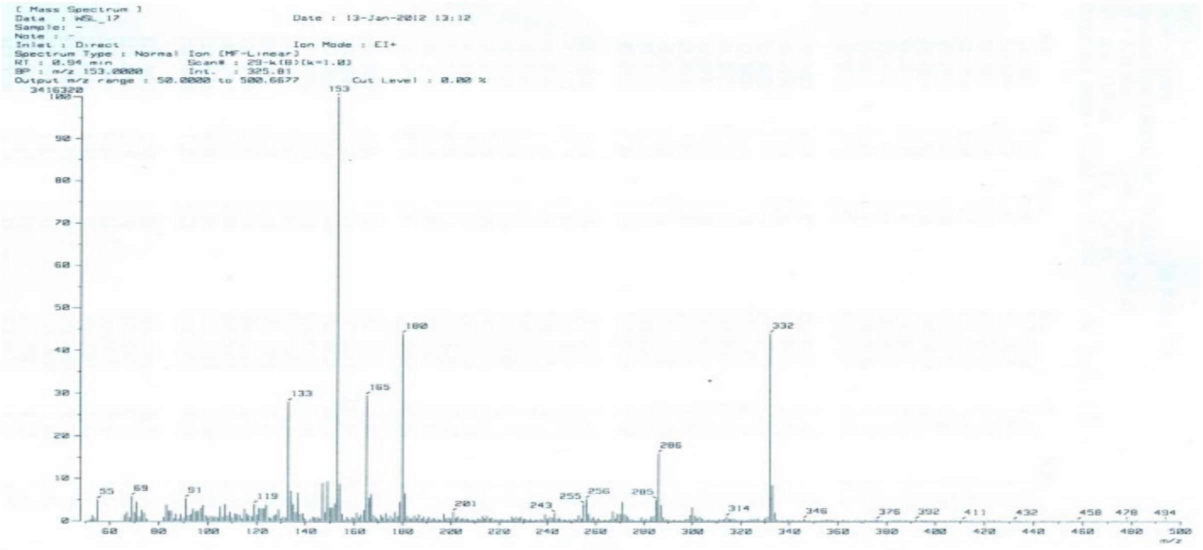

[Elemental Composition] Page: 1

Data : WSL_17 Date : 13-Jan-2012 13:52

Sample: -

Note : -

Inlet : Direct Ion Mode : EI+

RT : 0.85 min Scan#: 18

Elements : C 100/1, H 100/1, O 10/1

Mass Tolerance : 3mmu

Unsaturation (U.S.) : 0.0 - 30.0

Observed m/z	Int%	Err[ppm / mmu]	U.S.	Composition
332.0902	28.3	+1.9 / +0.6	10.0	C 17 H 16 O 7

Figure S27: EIMS and HREIMS data of compound **9**

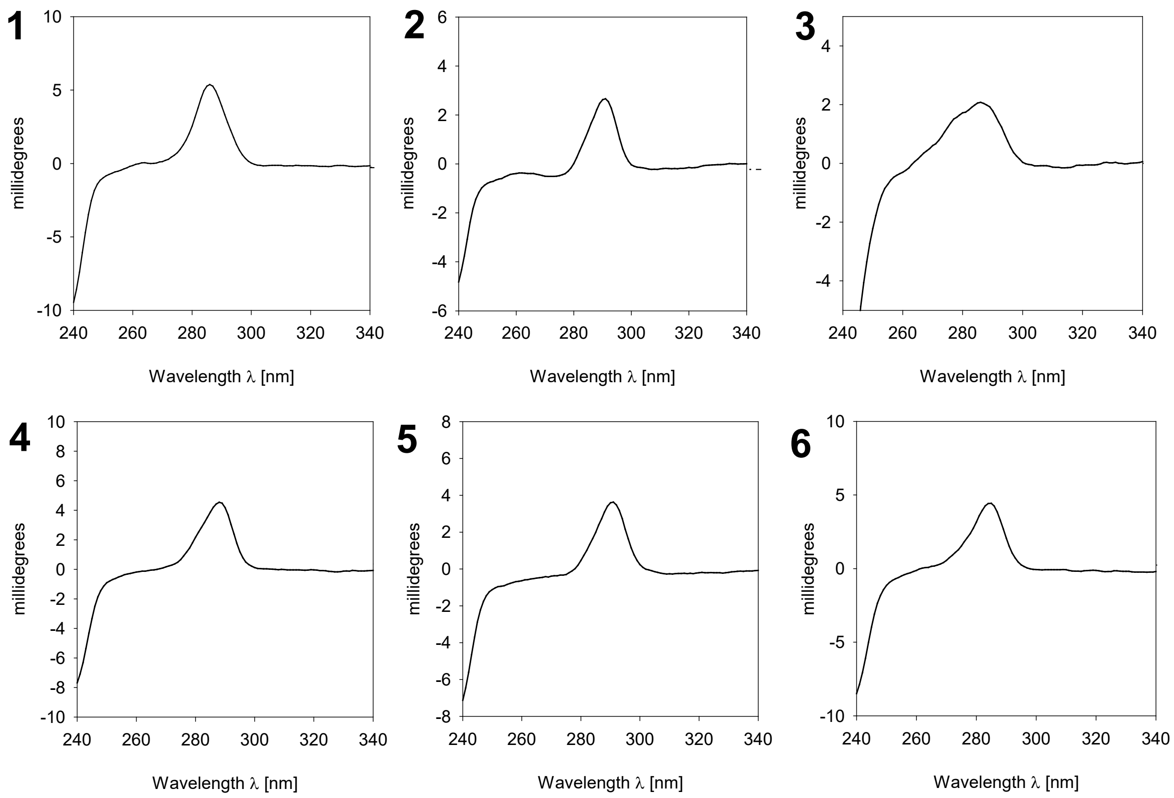

Figure S28: CD spectra of **1-6**

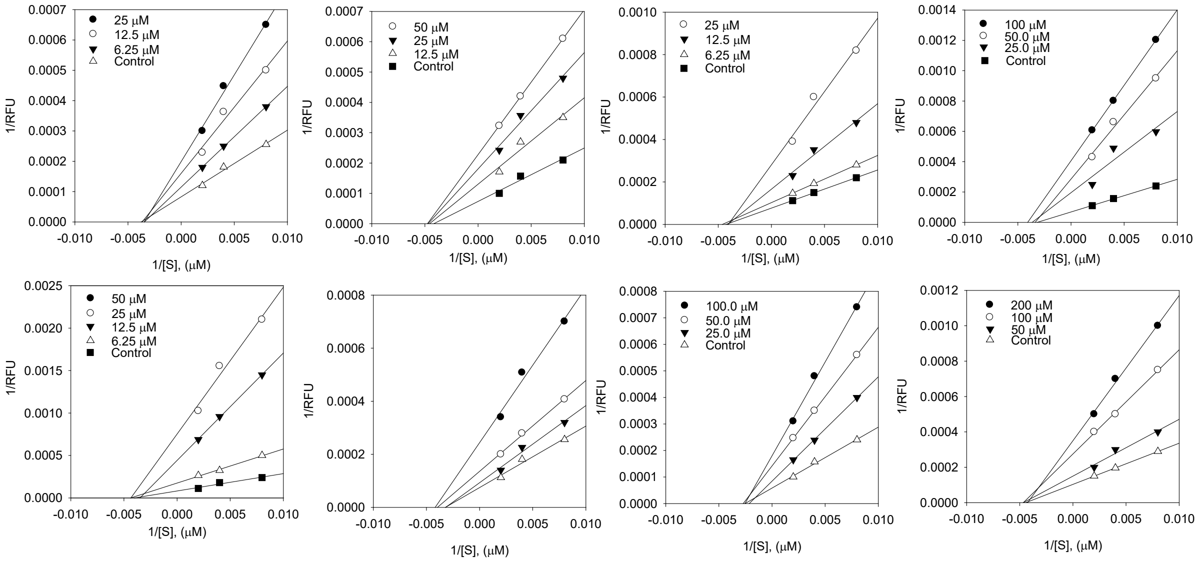

Figure S29: Lineweaver-Burk Plot for the inhibition of compounds

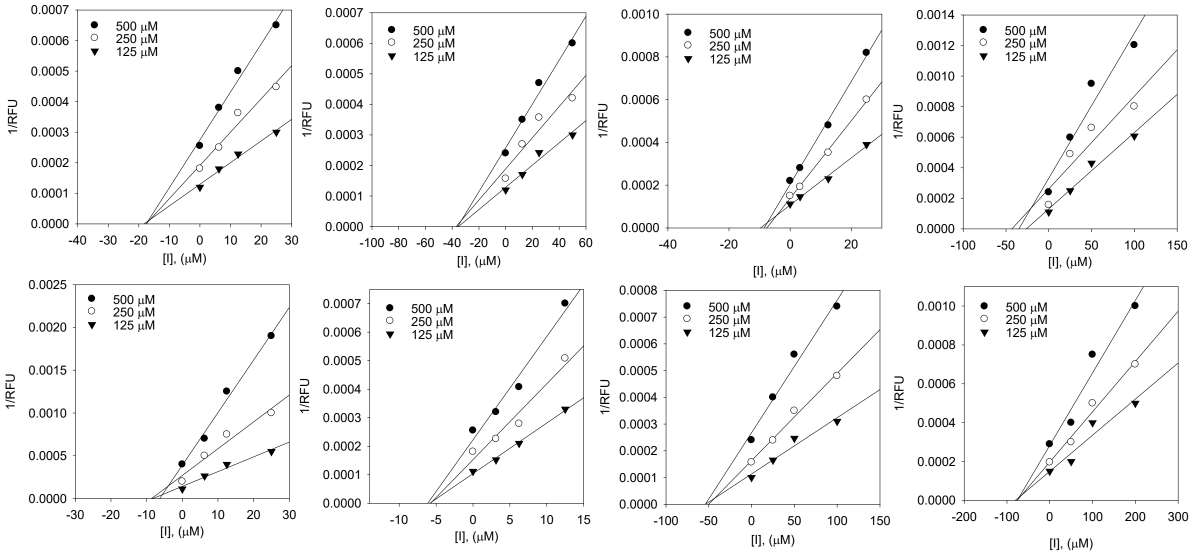

Figure S30: Dixon Plot for the inhibition of compounds

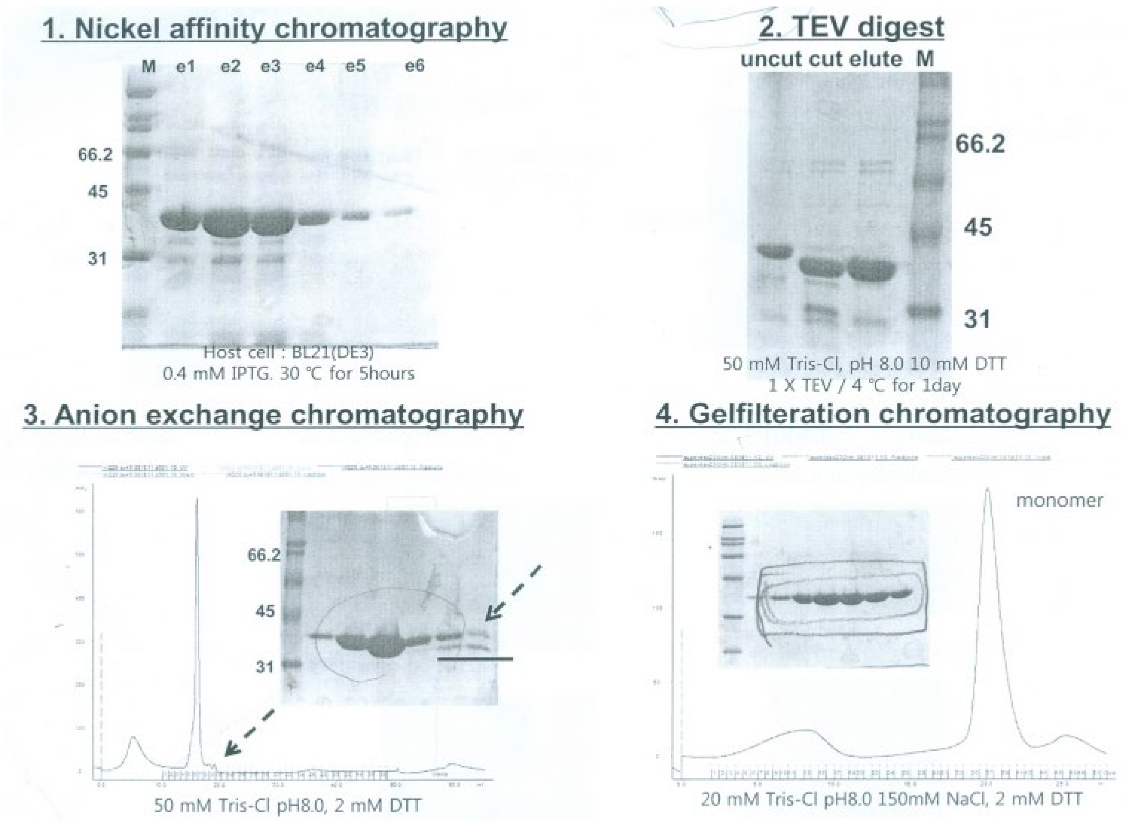

Figure S31: SARS-CoV PLpro purified from *E. Coli*

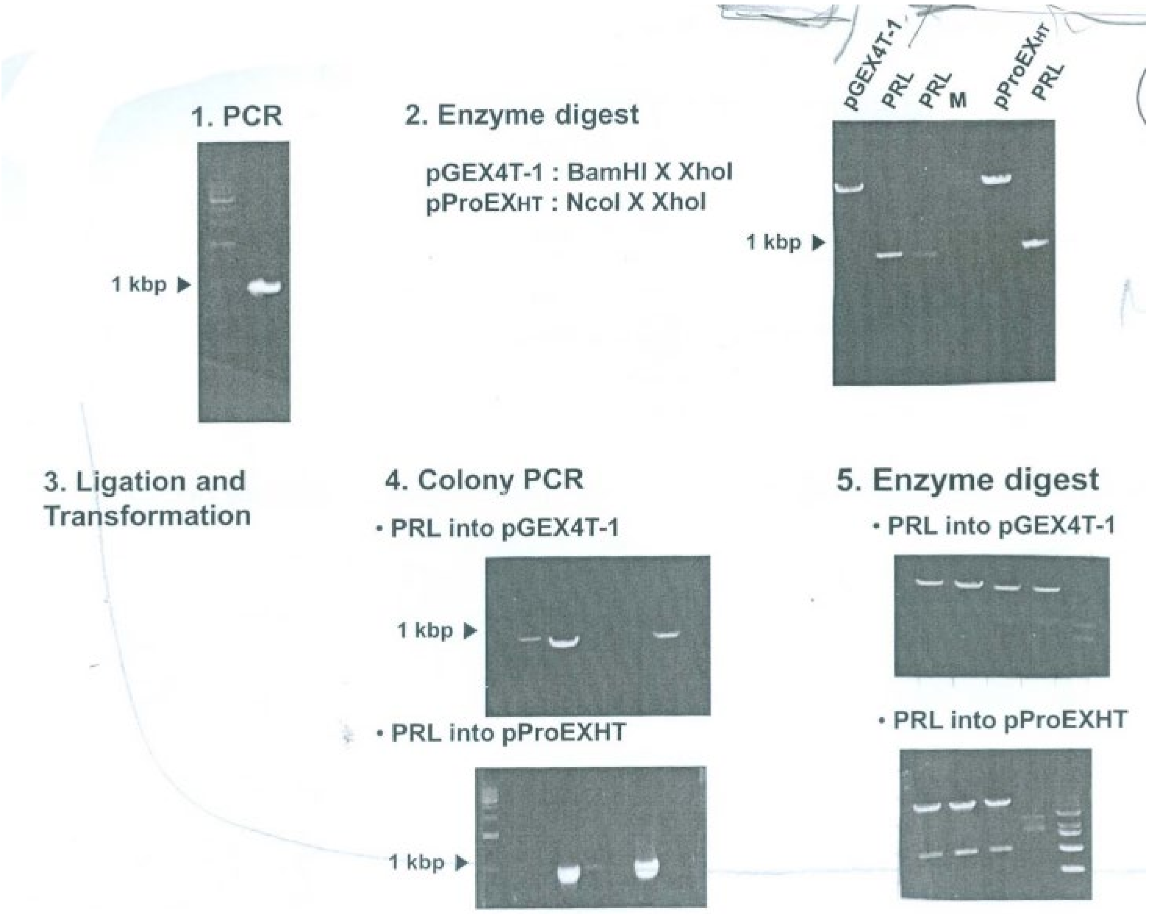

Figure S32: SARS-CoV PLpro purified from *E. Coli*
